# Troglitazone-Induced PRODH/POX-Dependent Apoptosis Occurs in the Absence of Estradiol or ERβ in ER-Negative Breast Cancer Cells

**DOI:** 10.3390/jcm10204641

**Published:** 2021-10-10

**Authors:** Sylwia Lewoniewska, Ilona Oscilowska, Thi Yen Ly Huynh, Izabela Prokop, Weronika Baszanowska, Katarzyna Bielawska, Jerzy Palka

**Affiliations:** 1 Department of Medicinal Chemistry, Medical University of Bialystok, Kilinskiego 1, 15-089 Bialystok, Poland; sylwialewoniewska123@wp.pl (S.L.); htyly79@gmail.com (T.Y.L.H.); izabela.prokop@umb.edu.pl (I.P.); w.baszanowska22@wp.pl (W.B.); katarzyna.bielawska@umb.edu.pl (K.B.); 2 Department of Analysis and Bioanalysis of Medicines, Medical University of Bialystok, Kilinskiego 1, 15-089 Bialystok, Poland; ilona.zareba@gmail.com

**Keywords:** apoptosis, PRODH/POX, collagen, estrogen, breast cancer cells

## Abstract

**Simple Summary:**

PRODH/POX (proline dehydrogenase/proline oxidase) is a mitochondrial enzyme that catalyzes proline degradation generating reactive oxygen species (ROS). Estrogens limit proline availability for PRODH/POX by stimulating collagen biosynthesis. It has been considered that estrogens determine efficiency of troglitazone (TGZ)-induced PRODH/POX-dependent apoptosis in breast cancer cells. The studies were performed in wild-type and PRODH/POX-silenced estrogen-dependent MCF-7 cells and estrogen-independent MDA-MB-231 cells. DNA and collagen biosynthesis were determined by radiometric method, ROS production was measured by fluorescence assay, protein expression was determined by Western blot and proline concentration by LC/MS analysis. We found that: i/TGZ-induced apoptosis in MDA-MB-231 occurs only in the absence of estradiol or ERβ, ii/the process is mediated by PRODH/POX, iii/and is facilitated by proline availability for PRODH/POX by TGZ-dependent inhibition of collagen biosynthesis (proline utilizing process). The data suggest that combined TGZ and anti-estrogen treatment could be considered in experimental therapy of ER negative breast cancers.

**Abstract:**

The impact of estradiol on troglitazone (TGZ)-induced proline dehydrogenase/proline oxidase (PRODH/POX)-dependent apoptosis was studied in wild-type and PRODH/POX-silenced estrogen receptor (ER) dependent MCF-7 cells and ER-independent MDA-MB-231 cells. DNA and collagen biosynthesis were determined by radiometric method, prolidase activity evaluated by colorimetric method, ROS production was measured by fluorescence assay. Protein expression was determined by Western blot and proline concentration by LC/MS analysis. PRODH/POX degrades proline yielding reactive oxygen species (ROS). Estrogens stimulate collagen biosynthesis utilizing free proline and limiting its availability for PRODH/POX-dependent apoptosis. TGZ cytotoxicity was highly pronounced in wild-type MDA-MB-231 cells cultured in medium without estradiol or in the cells cultured in medium with estradiol but deprived of ERβ (by ICI-dependent degradation), while in PRODH/POX-silenced cells the process was not affected. The TGZ cytotoxicity was accompanied by increase in PRODH/POX expression, ROS production, expression of cleaved caspase-3, caspase-9 and PARP, inhibition of collagen biosynthesis, prolidase activity and decrease in intracellular proline concentration. The phenomena were not observed in PRODH/POX-silenced cells. The data suggest that TGZ-induced apoptosis in MDA-MB-231 cells cultured in medium without estradiol or deprived of ERβ is mediated by PRODH/POX and the process is facilitated by proline availability for PRODH/POX by TGZ-dependent inhibition of collagen biosynthesis. It suggests that combined TGZ and antiestrogen treatment could be considered in experimental therapy of estrogen receptor negative breast cancers.

## 1. Introduction

Troglitazone is a member of thiazolidinediones (TZD), the new class of antidiabetic drugs [1,2] and agonist of peroxisome proliferator-activated receptor-γ (PPAR-γ) [3,4,5].

PPAR-γ is expressed in different types of cancer cells, including breast cancer cells. Activation of the receptor was found to attenuate cell growth and induce cell death [6]. Another PPAR-γ function is up-regulation of proline dehydrogenase/proline oxidase (PRODH/POX) expression. In the promoter sequence of the gene encoding PRODH/POX there are regions binding ligand-activated receptors, the so-called PPRE, or PPAR-γ response element [3,4].

We have come up with the assumption that the mechanism of PPAR-γ dependent apoptosis could involve PRODH/POX, a mitochondrial membrane associated enzyme that catalyzes proline degradation into Δ1-pyrroline-5-carboxylic acid (P5C). During this reaction, electrons are transferred via flavin adenine dinucleotide to cytochrome c in the respiratory chain producing ATP. However, when electrons are transferred directly to oxygen then the reactive oxygen species (ROS) are formed inducing apoptosis or autophagy [7,8,9]. Although the mechanism for switching from ATP to ROS production is not known, several factors have been implicated in this process [10,11]. One of them is prolidase, the enzyme supporting substrate for PRODH/POX. This enzyme catalyzes the last stage of collagen degradation, releasing proline from imidodipeptides. Proline could be also derived from amino acid metabolism, mainly glutamine, α-ketoglutarate and ornithine. However, the free proline may be reused for collagen biosynthesis limiting its availability for PRODH/POX. Therefore, collagen biosynthesis may affect PRODH/POX-dependent functions, including apoptosis. It is of interest that, in several cell types, estrogens are potent stimulators of collagen biosynthesis and cell growth [12,13,14,15].

Estrogen receptor (ER) activation is considered an important factor in breast cancer progression [16]. In fact, therapeutic efficacy of antiestrogen therapy is well-established by epidemiological data [17]. Studies on estrogen receptor (ER)-positive breast cancer cell lines (e.g., MCF-7 cells expressing α and β ER) provided evidence that estrogens stimulate proliferation of the breast cancer cells both in vitro and in vivo [18,19]. However, they are poorly metastatic and more responsive to antiestrogens, compared to ER negative breast cancer cells [20]. On the other hand, ER negative breast cancer cells, (e.g., MDA-MB-231, expressing only β estrogen receptor) are highly metastatic as established in rodent models [21]. It shows that estrogens play regulatory role in breast cancer cell growth and metastasis. The mechanism of estrogen function may involve multiple factors [22]. One of them is PPAR- γ that is capable of interacting with estrogen receptors [23]. It is a family member of nuclear hormone receptors, known as a ligand-activated transcription factor [6]. Natural PPAR-γ ligands are arachidonic acid metabolites and polyunsaturated fatty acids [24]. Synthetic ligands are represented by thiazolidinediones (TZD) the new class of antidiabetic drugs, e.g., troglitazone (TGZ), rosiglitazone, pioglitazone and ciglitazone [1,2]. The agonists in association with retinoid X receptor activate PPAR-γ that binds the complex to specific recognition sites of target genes inducing their expression [3,4,5].

PRODH/POX is also up-regulated by AMP-activated protein kinase (AMPK) [8]. This specific protein kinase is activated by phosphorylation when the AMP/ATP ratio rises, stimulating oxidative phosphorylation to restore normal ATP levels and inhibiting energy expenditure, such as cell proliferation [25,26]. Therefore, AMPK is regulated especially in conditions of energy shortage [26] in order to inhibit anabolic processes and stimulate catabolism which characterize cancer cells. Proline derived from protein degradation serves as an energy substrate in reaction catalyzed by PRODH/POX in mitochondria [7].

As TGZ induces PRODH/POX [27], and estrogens stimulate collagen biosynthesis [12,13,14,15] utilizing free proline and limiting its availability for PRODH/POX-dependent apoptosis, it has been considered that estrogen receptor status of breast cancer cells could determine ability of TGZ to induce PRODH/POX-dependent apoptosis.

**Scheme 1:**
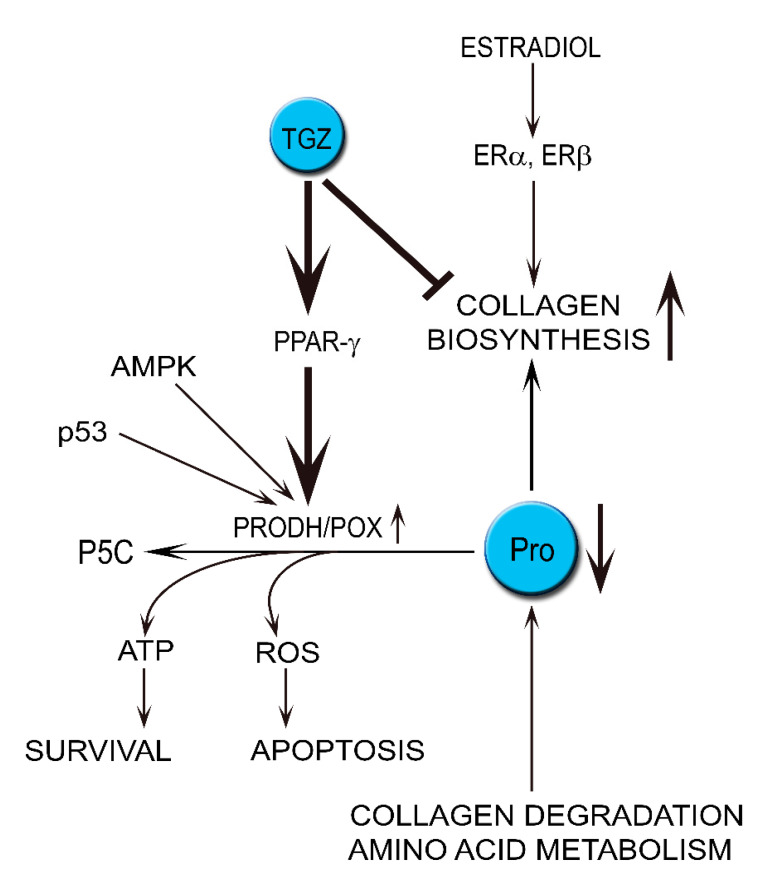
The hypothesis presents the role of estradiol in stimulation of proline utilization in collagen biosynthesis, limiting proline availability for PRODH/POX-dependent functions in breast cancer cells treated with troglitazone (TGZ), ligand of PPAR-γ inducing PRODH/POX expression.

The link between estrogens, collagen biosynthesis and degradation, PRODH/POX, PPAR-γ and apoptosis/survival led us to evaluate the impact of estrogen receptor activation on the above processes and PRODH/POX-dependent apoptosis in MCF-7 and MDA-MB-231 breast cancer cells as is outlined on the Scheme 1.

## 2. Materials and Methods

### 2.1. Cell Cultures

MCF-7 and MDA-MB-231 cells were obtained from ATCC (ATCC, Manassas, VA, USA). PRODH/POX-silenced MCF-7 and MDA-MB-231 cells were obtained as we descripted previously [28]. MCF-7 cells and MDA-MB-231 cells were maintained in DMEM without phenol red supplemented with 10% fetal bovine serum, 50 IU/mL penicillin, and 50 μg/mL streptomycin at 37 °C in a humidified atmosphere in the presence of 5% CO_2_. At about 80% of confluency the cells were treated for 24 h with estradiol (E, 2 nmol/L,), troglitazone (TGZ, 10 or 20 µmol/L) or both compounds in DMEM without phenol red supplemented with 10% CPSR1.

### 2.2. DNA Biosynthesis Assay

DNA biosynthesis was evaluated by [methyl-^3^H]-thymidine incorporation into DNA as described previously [29]. The cells were cultured in 24-well plate to 80% of confluency. After that they were incubated in medium with or without estradiol (E) and troglitazone (TGZ) for 24 h with 0.5 μCi/mL of [methyl-^3^H]-thymidine. Incorporation of the tracer into DNA was measured by Liquid Scintillation Analyzer Tri-Carb 2810 TR and calculated using Quanto Smart TM software.

### 2.3. Collagen Biosynthesis

Collagen biosynthesis was evaluated by 5[^3^H]-proline (5 μCi/mL) incorporation into proteins susceptible to bacterial collagenase. The cells were cultured in the presence of tracer in medium with or without estradiol (E) and troglitazone (TGZ) for 24 h. Incorporation of tracer into collagen was measured in accordance to the method of Peterkofsky et al. [30]. Incorporation of radioactive proline was detected by Liquid Scintillation Analyzer Tri-Carb 2810 TR and calculated using Quanto Smart TM software. Results are shown as combined values for cell plus medium fractions.

### 2.4. Determination of Prolidase Activity

The activity of prolidase was determined according to the method of Myara et al. [31]. The cells were cultured in 10 cm diameter dishes and incubated in medium with or without estradiol (E) and troglitazone (TGZ) for 24 h. Protein concentration was measured by the method of Lowry et al. [32]. Enzyme activity was reported as nanomoles of proline released from synthetic substrate (glycyl-proline), during 1 min per milligram of supernatant protein of cell homogenate.

### 2.5. Western-Immunoblot Analysis

Protein analysis was performed by Western blot as previously described [28,29]. Cell lysates of the cells were harvested and subjected to SDS-PAGE in 10% polyacrylamide gel (1 h, 125 V, room temperature (RT)). The protein was transferred to 0.22 μm pore-sized nitrocellulose (wet transfer, 1 h, 100 mA, RT). After the transfer, membranes were blocked with 5% non-fat dry milk in TBS-T (20 mmol/L Tris–HCl, 150 mmol/L NaCl, 0.05% Tween 20, pH 7.4) and incubated with rabbit anti-cleaved-caspase-9, anti-cleaved-caspase-3, anti-p53, anti-PARP, anti-cleaved-PARP, anti-AMPK alpha, anti-PPAR gamma, anti-GAPDH and goat-anti-PRODH, diluted 1:1000 in blocking buffer. Then membranes were washed in TBS with 0.05% Tween (TBST) 3 × 15 min and incubated with respective HRP-linked secondary antibody at concentration 1:7500 for 60 min at RT with gentle agitation. After washing in TBS-T (5 × 5 min) membranes were incubated with Amersham ECL Western Blotting Detection Reagent. Pictures were taken using BioSpectrum Imaging System UVP. Blots (done in three repeats) and densitometry are contained in Supplementary Data (Supplementary Figures S5–S18).

### 2.6. LC–MS-Based Quantitative Analysis

Proline concentration was measured according to the method of Klupczynska et al. [33]. Briefly, cells were analyzed by Agilent 1260 Infinity HPLC system coupled to Agilent 6530 Q-TOF mass spectrometry detector with electrospray ionization (Agilent Technologies, Santa Clara, CA, USA) as an ion source in positive ionization mode. Samples were injected onto a HILIC column (Luna HILIC, 2 × 100 mm, 3 μm, Phenomenex, Torrance, CA, USA) thermostated at 30 °C. Protein concentration was used to normalize the obtained results. The data was presented as a percent of the control value.

### 2.7. ROS Generation Assessment

Intracellular reactive oxygen species accumulation was measured using DCFH-DA as a fluorescent probe. Briefly, cells were pre-incubated with DCFH-DA (20 µM) in culture medium for 30 min, washed twice with PBS and treated with increasing concentrations of for 24 h with estradiol (E, 2 nmol/L,), troglitazone (TGZ, 10 or 20 µmol/L) or both compounds in DMEM without phenol red. The fluorescent intensity was measured at excitation/emission wavelength of 488/535 nm using TECAN Infinite^®^ M200 PRO (Männedorf, Switzerland). The results were presented as a percent of the control value.

### 2.8. Statistical Analysis

**Figure 1:**
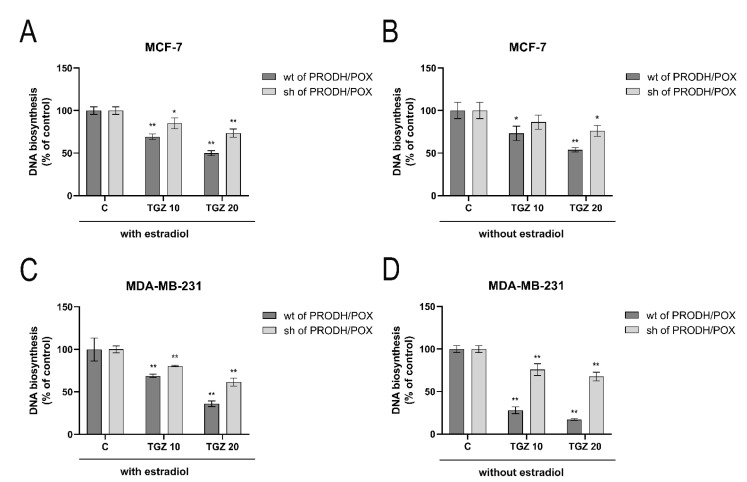
DNA biosynthesis in wild type (wt of PRODH/POX) and PRODH/POX-silenced (sh of PRODH/POX) MCF-7 and MDA-MB-231 cells treated for 24 h with troglitazone (TGZ) in the presence (**A**,**C**) and absence (**B**,**D**) of estradiol. The mean values ± standard deviation (SD) from 3 experiments done in duplicates are presented. Asterisks (*) indicate statistical differences between studied cells compared to controls at * *p* < 0.01 and ** *p* < 0.001.

**Figure 2:**
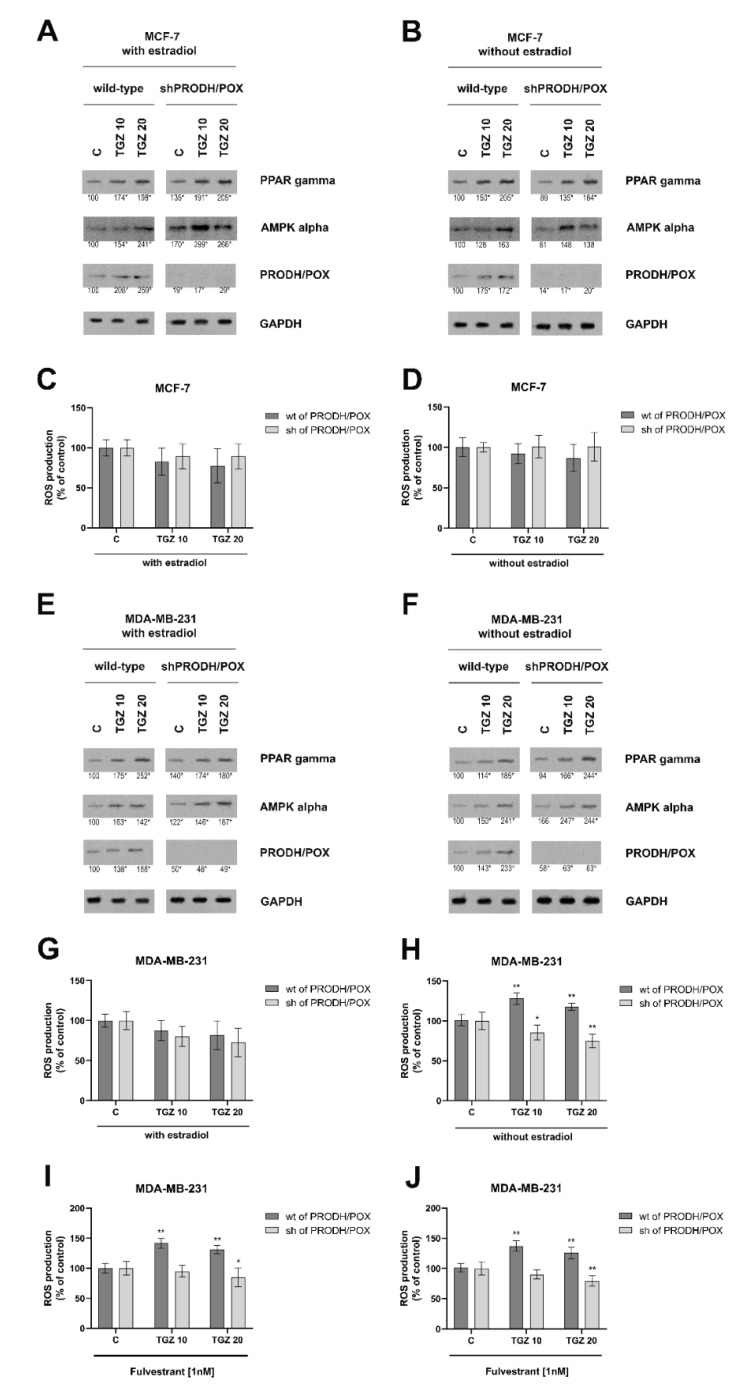
PRODH/POX, AMPK and PPAR-γ expressions in wild type (wt of PRODH/POX) and PRODH/POX-silenced (sh of PRODH/POX) MCF-7 and MDA-MB-231 cells treated for 24 h with troglitazone (TGZ) in the presence (**A**,**E**) and absence (**B**,**F**) of estradiol. Representative blot images were shown (the mean value of densitometric analysis of protein bands presented below each blot; * *p* < 0.01). Supplementary Materials contain statistical analysis of the evaluated proteins (Supplementary Material, Supplementary Figures S2–S7). ROS production is presented in wild type (wt of PRODH/POX) and PRODH/POX-silenced (sh of PRODH/POX) MCF-7 and MDA-MB-231 cells cultured with TGZ and/or fulvestrant (ICI 182 780) in the presence (**C**,**G**,**I**) and absence (**D**,**H**,**J**) of estradiol. The mean values ± standard deviation (SD) from 3 experiments done in duplicates are presented. Asterisks (*) indicate statistical differences between studied cells compared to controls at * *p* < 0.01 and ** *p* < 0.001.

In the experiments presented in Figure 1 and Figure 2, the mean values for six assays ± standard deviations (SD) were calculated. The results were submitted to statistical analysis using the Shapiro–Wilk test and Kolmogorov–Smirnov tests. All results have a normal distribution. To assess statistical significance in conducted experiments, one-way ANOVA with Dunnett’s multiple comparison test with 99% confidence interval was used (GraphPad PRISM v5.0, GraphPad Software Inc., San Diego, CA, USA). Results were considered significant at *p* < 0.01 level and are denoted by an asterisk (*).

## 3. Results

### 3.1. The Design of Experiments

ER-positive, MCF-7 cells (expressing α and β estrogen receptor), wild-type (wt of MCF-7 cells) and PRODH/POX-silenced (shPRODH/POX MCF-7 cells), and ER-negative, MDA-MB 231 cells (expressing only β estrogen receptor), wild-type (wt of MDA-MB-231 cells) and PRODH/POX-silenced (shPRODH/POX MDA-MB-231 cells), were used to study the effect of estrogen receptor activation on the PRODH/POX-dependent apoptosis and related processes. Preparation of PRODH/POX-silenced MCF-7 and MDA-MB-231 cells were described previously [28,29]. The representative blots with efficiency of stably silenced PRODH/POX are presented in Supplementary Materials (Supplementary Figure S1). PRODH/POX-dependent functions play critical roles in proline availability that is regulated by prolidase activity (proline supporting enzyme) and collagen biosynthesis (proline utilizing process). These processes are estrogen-dependent [12,13,14,15,34]. We used medium without phenol red, containing 10% CPSR1 and studied the effect of 2 nM estradiol on DNA biosynthesis, ROS production, proline concentration, collagen biosynthesis, prolidase activity and expression of apoptosis markers. As an inductor of PRODH/POX expression we used troglitazone (TGZ), at final concentrations of 10 and 20 µM, as previously established [34].

### 3.2. PRODH/POX and β-Estrogen Receptor (ERβ) Participate in TGZ-Dependent Inhibition of DNA Biosynthesis in MCF-7 and MDA-MB-231 Cells

TGZ inhibits DNA biosynthesis in both MCF-7 (Figure 1A,B) and MDA-MB-231 (Figure 1C,D) cells were cultured in the presence (Figure 1A,C) and absence (Figure 1B,D) of estradiol. The inhibition was partially dependent on PRODH/POX, as in the PRODH/POX-silenced cells the process was much less pronounced. However, in wild type MDA-MB-231 cells cultured in medium without estradiol, TGZ strongly inhibited DNA biosynthesis, while in PRODH/POX silenced cells the process was much less affected. It suggests that PRODH/POX and/or β-estrogen receptor (ERβ), that is expressed in MDA-MB-231 cells may participate in TGZ-dependent inhibition of DNA biosynthesis in these cells.

### 3.3. PRODH/POX and β-Estrogen Receptor (ERβ) Are Involved in TGZ-Dependent ROS Production in Breast Cancer Cells

TGZ induces PRODH/POX expression in both wild type breast cancer cells independently of the presence or absence of estradiol (Figure 2A,B). In PRODH/POX silenced breast cancer cells the enzyme was not detected and TGZ did not affect its expression. However, TGZ in a dose dependent manner induced expression of PPAR-γ and AMPK α in wild type and PRODH/POX-silenced MCF-7 as well as MDA-MB-231 cells cultured in the presence or absence of estradiol (Figure 2A,B,E,F).

TGZ did not significantly affect ROS production in both lines of MCF-7 cells cultured in the presence or absence of estradiol (Figure 2C,D) and in wild type MDA-MB-231 cells cultured in medium with estradiol (Figure 2G). However, in PRODH/POX-silenced MDA-MB-231 cells, ROS production was significantly decreased independently of the presence or absence of estradiol (Figure 2G,H). It suggests that PRODH/POX is involved in ROS production in PRODH/POX expressing breast cancer cells. Interestingly, in wild type of MDA-MB-231 cells cultured in medium without estradiol ROS production was significantly increased (Figure 2H). As MDA-MB-231 cells express only ERβ it could suggest that ERβ may participate in TGZ-dependent generation of ROS in wild type MDA-MB-231 cells cultured in medium without estradiol. However, when ERβ was removed from MDA-MB-231 cells by ICI-182-780 (fulvestrant)-dependent degradation, TGZ regardless on the absence or presence of estradiol in the medium also induced ROS production, suggesting that ERβ is not required for the process (Figure 2I,J).

### 3.4. TGZ-Dependent Apoptosis in Breast Cancer Cells Is More Pronounced in Wild Type MDA-MB-231 Cells Cultured in Estradiol Free Medium than in MCF-7 Cells Cultured in the Same Conditions

**Figure 3:**
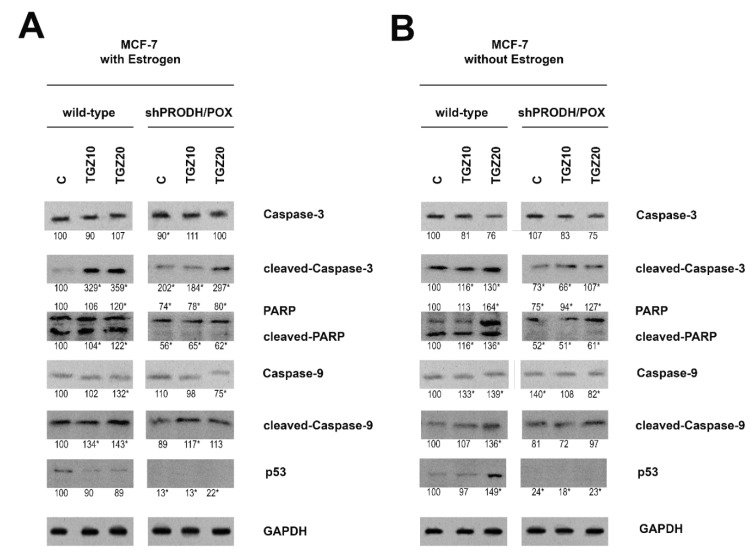
Western blot for cleaved caspase-3, PARP, caspase-9 and p53 in wild type and PRODH/POX-silenced (shPRODH/POX) MCF-7 cells treated with troglitazone (TGZ) for 24 h in the presence (**A**) and absence (**B**) of estradiol. GAPDH expression was used as a loading control. Representative blot images were shown (the mean value of densitometric analysis of protein bands presented below each blot; * *p* < 0.01). Supplementary Materials contain statistical analysis of the evaluated proteins (Supplementary Material, Supplementary Figures S8–S13).

**Figure 4:**
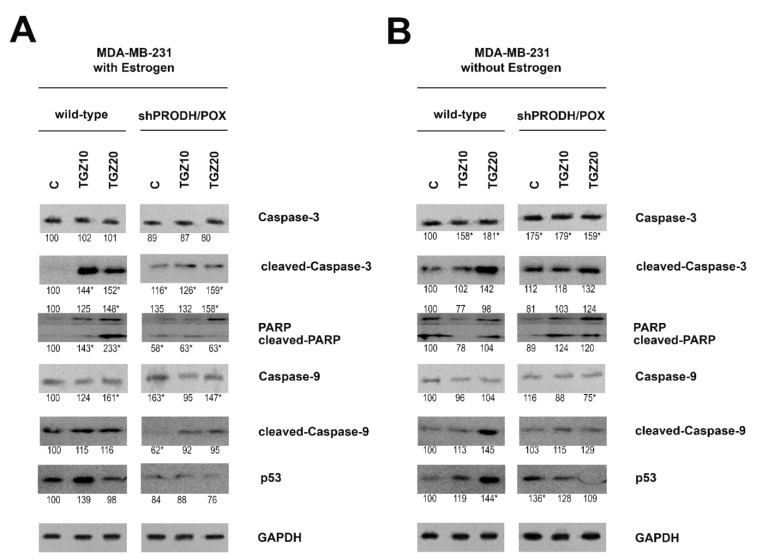
Western blot for cleaved caspase-3, PARP, caspase-9 and p53 in wild type and PRODH/POX-silenced (shPRODH/POX) MDA-MB-231 cells treated with troglitazone (TGZ) for 24 h in the presence (**A**) and absence (**B**) of estradiol. GAPDH expression was used as a loading control. Representative blot images were shown (the mean value of densitometric analysis of protein bands presented below each blot; * *p* < 0.01). Supplementary Materials contain statistical analysis of the evaluated proteins (Supplementary Material, Supplementary Figures S14–S19).

TGZ induced expression of cleaved caspase-3, caspase-9 and PARP in both cell lines (MCF-7 and MDA-MB-231) cultured in the presence or absence of estradiol (Figure 3A,B; Figure 4A,B). However, their expressions were more pronounced in wild type of MDA-MB-231 cells (Figure 4B) cultured in estradiol free medium, compared to the cells cultured in medium with estradiol. Increase in the expression of studied caspases in TGZ-treated cells was accompanied by increase in the expression of p53 particularly in MDA-MB-231 cells (Figure 4B) cultured in estradiol free medium.

It suggests that TGZ-dependent apoptosis in breast cancer cells is highly pronounced in ERβ expressing MDA-MB-231 cells cultured in estradiol free medium.

### 3.5. TGZ Contributes to the Increase in Proline Availability for PRODH/POX via down Regulation of Collagen Biosynthesis and Up-Regulation of Prolidase Activity in Breast Cancer Cells

**Figure 5:**
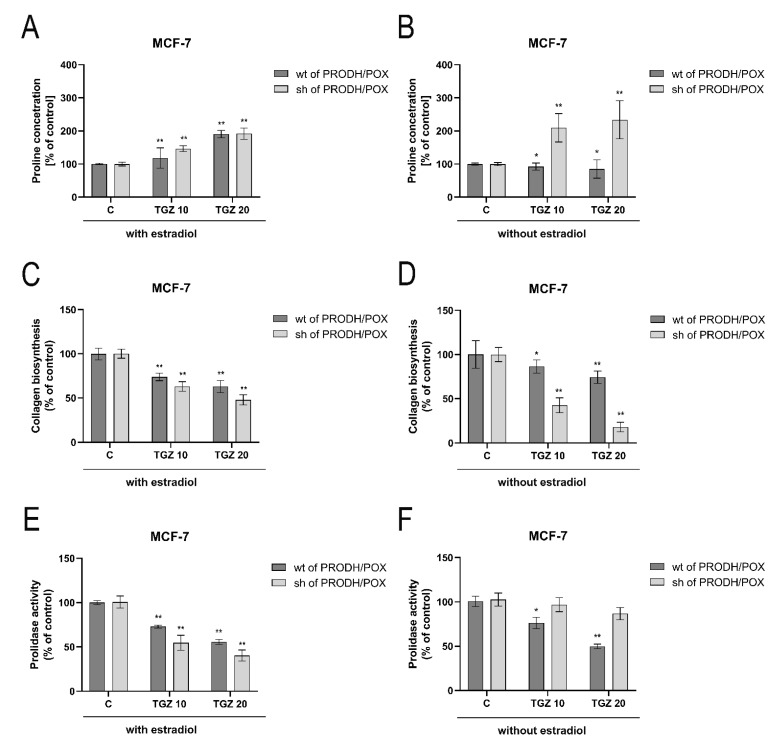
Proline concentration (**A**,**B**), collagen biosynthesis (**C**,**D**) and prolidase activity (**E**,**F**) in wild type (wt of PRODH/POX) and PRODH/POX-silenced (sh of PRODH/POX) MCF-7 cells stimulated by troglitazone (TGZ) for 24 h in the presence (**A**,**C**,**E**) and absence (**B**,**D**,**F**) of estradiol. The mean values ± standard deviation (SD) from 3 experiments done in duplicates are presented. Asterisks (*) indicate statistical differences between studied cells compared to controls at * *p* < 0.01 and ** *p* < 0.001.

PRODH/POX-induced apoptosis (through ROS generation) in breast cancer cells is dependent on proline availability. Intracellular free proline content is regulated mainly by collagen biosynthesis (proline utilizing process) and prolidase activity (proline releasing enzyme). In wild type and PRODH/POX-silenced MCF-7 cells cultured in medium containing estradiol, TGZ induced dose-dependent increase in proline concentration (Figure 5A) and inhibition of collagen biosynthesis (Figure 5C) and prolidase activity (Figure 5E). TGZ-dependent increase in proline concentration and decrease in collagen biosynthesis and prolidase activity were more pronounced in PRODH/POX silenced cells. In MCF-7 cells cultured in medium without estradiol TGZ contributed to increase in proline concentration and decrease in collagen biosynthesis in PRODH/POX-silenced cells while in wild type MCF-7 cells the processes were much less affected (Figure 5B,D). However, TGZ inhibited prolidase activity in wild type MCF-7 cells, while it had no significant effect on the enzyme activity in PRODH/POX silenced cells cultured in estradiol free medium (Figure 5F).

**Figure 6:**
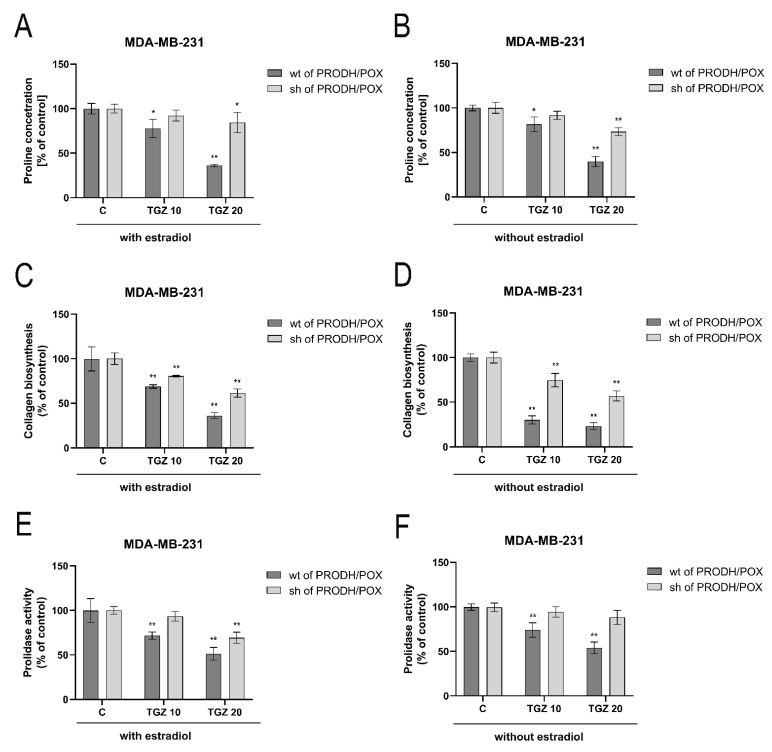
Proline concentration (**A**,**B**), collagen biosynthesis (**C**,**D**) and prolidase activity (**E**,**F**) in wild type (wt of PRODH/POX) and PRODH/POX-silenced (sh of PRODH/POX) MDA-MB-231 cells stimulated by troglitazone (TGZ) for 24 h in the presence (**A**,**C**,**E**) and absence (**B**,**D**,**F**) of estradiol. The mean values ± standard deviation (SD) from 3 experiments done in duplicates are presented. Asterisks (*) indicate statistical differences between studied cells compared to controls at * *p* < 0.01 and ** *p* < 0.001.

In both wild type and PRODH/POX-silenced MDA-MB-231 cells cultured in medium containing estradiol, TGZ contributed to decrease in proline concentration (Figure 6A), collagen biosynthesis (Figure 6B) and prolidase activity (Figure 6C). The inhibition was less pronounced in PRODH/POX-silenced cells. In wild type MDA-MB-231 cells cultured without estradiol, TGZ contributed to dose-dependent decrease in proline concentration (Figure 6B), inhibition of collagen biosynthesis (Figure 6D) and prolidase activity (Figure 6F), while in PRODH/POX silenced cells, proline concentration and prolidase activity were not significantly affected and the inhibition of collagen biosynthesis was less pronounced than in MDA-MB-231 wild type cells (Figure 6B,D,F). It suggests that TGZ-induced inhibition of collagen biosynthesis could facilitate proline availability for PRODH/POX-dependent functions.

## 4. Discussion

This study investigated the role of estrogen receptor activation on troglitazone (TGZ)-induced PRODH/POX -dependent apoptosis in four models of breast cancer cells. Although cell line models have some limitations (e.g., inability to observe systemic phenomena), they are a powerful tool which offer several advantages. Certainly, the cell models allow strict control of the conditions of the experiment in order to establish the critical factor affecting the studied processes. They are especially helpful in case of limited availability of clinical samples or in vivo models (e.g., estradiol deficiency or estrogen receptor status). Therefore, results on cell models allow to predict the consequences of pharmacotherapeutic manipulation in human. Different treatment regimens and combinations of therapies have been tested using cell lines which have yielded interesting and potentially promising results that currently have an application value [35,36]. In this study we present the result on four cell lines: estrogen-dependent MCF-7 and estrogen-independent MDA-MB-231 cell line and their respective PRODH/POX-silenced cell lines, which were described previously [28,29,37].

The data presented in this report suggest that TGZ-induced PRODH/POX-dependent apoptosis is conditioned by the status of the ERs in breast cancer cells. In MDA-MB-231 cells cultured in medium without estradiol or deprived of ERβ, TGZ induces PRODH/POX-dependent apoptosis and the process is facilitated by proline availability for PRODH/POX by TGZ-dependent inhibition of collagen biosynthesis. The underlying mechanism involves activation of PRODH/POX (by PPAR-γ ligand, TGZ) that under availability of proline (substrate for PRODH/POX) and dependently on the ER status (absence of estradiol or ERβ) induces apoptosis in ER negative breast cancer cells. The role of PRODH/POX in this process was confirmed by experiments showing that in PRODH/POX silenced cells apoptosis does not occur. The role of proline availability for PRODH/POX-dependent functions was well established [38]. Although estrogens stimulate collagen biosynthesis [12,13,15] limiting proline availability for PRODH/POX-dependent apoptosis, in the absence of estradiol and presence of TGZ the process (collagen biosynthesis) is inhibited leading to increase in the availability of substrate (proline) for PRODH/POX. We have found that in MDA-MB-231 cells cultured in medium without estradiol, TGZ contributed to apoptotic phenotype of breast cancer cells, as detected by increase in active caspase-3, -9 and PARP expressions. The effect was not found in MCF-7 cells, independently of the presence or absence of estradiol, and in MDA-MB-231 cells cultured in the medium with estradiol. The mechanism for the process was found at the level of collagen biosynthesis that is up-regulated by estrogens [27,34,39].

Collagen biosynthesis is the most effective process in utilization of intracellular proline, substrate for PRODH/POX. Previously we found that PPAR-γ ligands evoke collagen biosynthesis inhibiting activity [34]. It was proved in the present study. Such an activity of TGZ supports proline for PRODH/POX-dependent functions. However, estrogens and estrogen receptor status seem to play critical role in this process. In ER positive, wild type breast cancer MCF-7 cells treated with TGZ, the expression of PRODH/POX was increased independently of the presence or absence of estradiol, while ROS production and expression of apoptosis markers were not affected. The similar effect was found in estrogen receptor negative MDA-MB-231 cells, cultured in medium with estradiol. However, in MDA-MB-231cells, cultured in medium without estradiol, pro-apoptotic potential of TGZ was pronounced. The same effect was achieved in the cells cultured in the presence of estradiol but deprived ERβ by fulvestrant treatment. It suggests that ERβ may participate in the inhibition of PRODH/POX-dependent ROS generation in breast cancer cells. The explanation for the phenomenon is based on the fact, that estrogens activate collagen biosynthesis [13,15,34] that utilizes proline, substrate for PRODH/POX-dependent apoptosis. Removal of either ERβ or estradiol eliminate their role in stimulation of collagen biosynthesis, making proline available for PRODH/POX-dependent functions. In fact, it has been previously suggested that collagen biosynthesis is stimulated by ERβ [40] while inhibition of collagen biosynthesis induces PRODH/POX-dependent apoptosis in breast cancer cells [28].

However, in contrast to these results, it has been suggested that activation of ERβ (expressed in ER negative MDA-MB-231 cells) contributes to pro-apoptotic phenotype of breast cancer cells, while activation of ERα (expressed in ER positive MCF-7 cells) induces anti-apoptotic effects [41]. It is partially corroborated by studies showing that ER positive breast cancer cells are less metastatic, compared to ER negative ones [42,43]. In this context MDA-MB-231 cells expressing only ERβ are more invasive than MCF-7 cells, expressing both ERs. However, it is in contrast to pro-apoptotic potential of ERβ expressing cells.

It cannot be excluded that pro-apoptotic phenotype of TGZ-treated breast cancer cells, cultured in the absence of estradiol is due to other mechanisms. Some studies documented that there is a cross talk between ERs and PPAR-β [44], or ERs and P53 [41]. In such a case, estradiol could compete for binding site in ERs, therefore, the effects of ERs were seen only in the absence of estradiol. Such a mechanism was established previously in case of ER-dependent regulation of collagen biosynthesis, where ER was removed by Fulvestrant (ICI 182-780)-induced proteasomal degradation in MDA-MB-231 cells [45]. In view of estrogen-dependent modulation of collagen biosynthesis [34,46,47] it cannot be excluded that ER/PPAR-γ cross-talk regulates proline availability for PRODH/POX-dependent functions. However, this hypothesis requires to be explored.

The absence of estradiol or ERβ in the cell culture is critical requirement for TGZ-induced PRODH/POX-dependent apoptosis in MDA-MB-231 cells. Probably, in vivo such a situation never happens, as estradiol in tissues is ubiquitous. This finding may be of importance in the experimental therapy of ER negative breast cancers by combined use of TGZ and anti-estrogens. In fact, it has been well established that estrogen receptor status determine efficacy of breast cancer therapy and its determination has predictive and prognostic value, particularly in triple-negative breast cancer [48]. However, in triple-negative breast cancer cells, MDA-MB-231, TGZ did not induce apoptosis [49]. Similarly, TGZ does not affect the viability of ER positive MCF-7 cells, however, it inhibits invasion of the cells [50]. In respect to ER positive breast cancer cells, PPAR-γ ligands as TGZ have a therapeutic limitation because it has been established that ERα blocks PPAR-γ signaling. Blocking ER by tamoxifen was shown to counteract ERα-mediated inhibition of PPAR-γ function. On the other hand, activation of ERα by estradiol blocked TGZ- induced PPAR-γ -dependent cell cycle arrest, indicating the resistance of ERα-positive breast cancer cells to TGZ. Based on these data it has been concluded that combination of troglitazone with tamoxifen may represent therapeutic approach to growth inhibition of ERα-positive MCF-7 cells [50]. It is not known whether a similar phenomenon occurs in MDA-MB-231 cells. Therefore, further studies on antiestrogen therapy accompanied by TGZ treatment should be undertaken as an approach to experimental therapy of ER negative breast cancer cells.

Downregulation of AMPK, metabolic regulator involved in control of cell growth and survival has been established as a major contributor to carcinogenesis in many types of human cancer [51]. We have found that TGZ induced in dose-dependent manner AMPK expression. Of interest is observation that TGZ induced AMPK also in PRODH/POX-silenced cells. Although AMPK is potent stimulator of PRODH/POX [27] it did not stimulate the enzyme expression in PRODH/POX silenced cells. It seems that the efficiency of this stimulation depends on constitutive level of PRODH/POX expression.

Another interesting factor in studies on PRODH/POX dependent apoptosis is p53 protein. This transcription factor is the best characterized apoptosis inducing factor and also the most potent factor regulating expression of PRODH/POX. The presence of response element for p53 protein in the promoter sequence of the gene coding PRODH/POX has been demonstrated. It shows direct participation of p53 in the transcription of PRODH/POX [11]. However, in MDA-MB-231 cells it has probably low importance due to mutation [52,53] that eliminate the transcription factor as a player in the mechanism driving PRODH/POX-dependent apoptosis in ER negative breast cancer cells. Nevertheless, p53 expression was up-regulated in TGZ-treated breast cancer cells cultured in estradiol-free medium. Interestingly, a pro-apoptotic effect of AMPK in cancer cells and a mutual relationship between AMPK and p53 have been reported. It cannot be excluded that pro-apoptotic effect of TGZ involves cross-talk between AMPK and p53 [51]. Although the mechanism is not well understood, the activation of p53 in the cells cultured in estradiol-free medium is supported by some other authors, suggesting that estradiol inactivates P53 [54] or estrogen receptor prevents p53-dependent apoptosis in breast cancer [55,56]. It seems that in the absence of estradiol, TGZ-treated MCF-7 cells may undergo apoptosis by p53 signaling [50,51], while MDA-MB-231 cells, due to p53 mutation, through PRODH/POX-dependent ROS generation.

Presently, the selective ER modulator, tamoxifen, is the only endocrine agent with approval for prevention and treatment of ER positive breast cancer [57]. In view of the presented results it would be reasonable to perform more clinical studies on tamoxifen treated ER negative breast cancer cells. Although the cells lack ERα, the expression of ERβ is sufficient to trigger estrogen-responsivity via ERα-independent pathways [58]. It has been reported that estrogens promote the brain metastatic colonization of TNBC cells [59] while ovariectomy decreased the frequency of brain metastases as compared to estrogen supplementation, and that the combination of ovariectomy and aromatase inhibitor further reduced the frequency of large lesions to 14% of the estrogen control. Furthermore, it was demonstrated [60] that increasing levels of circulating estrogens was sufficient to promote the formation and progression of ERα-negative cancers. These data suggest that endocrine therapy options directed against ERβ and estrogens should be considered for treatment of ER negative breast cancer.

## 5. Conclusions

**Scheme 2:**
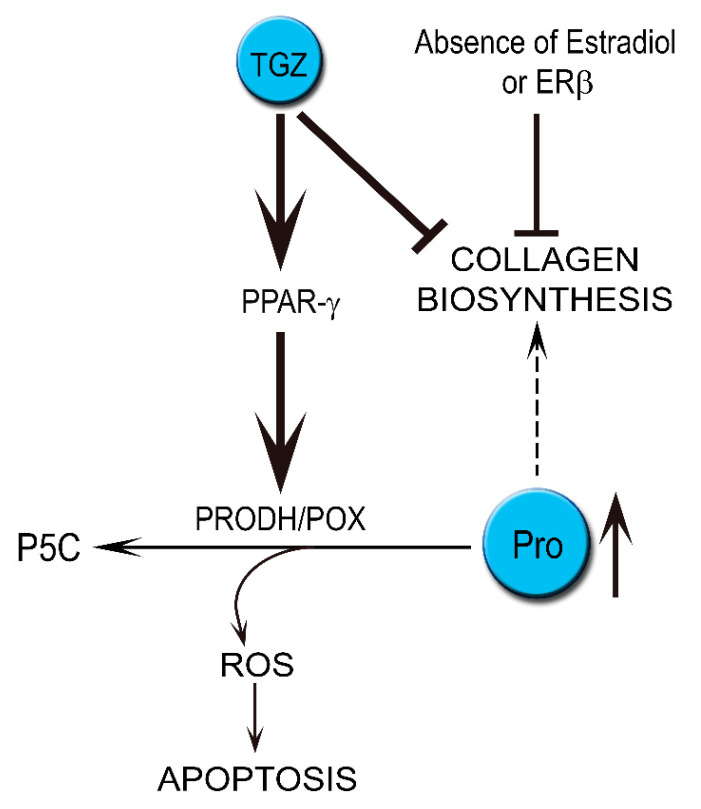
The role of ER status (presence or absence of estradiol or ERβ) in the mechanism of TGZ-induced PRODH/POX-dependent apoptosis in breast cancer MDA-MB-231 cells. ER—estrogen receptor; P5C—Δ1-pyrroline-5-carboxylic acid; PPAR-γ—peroxisome proliferator-activated receptor γ; Pro—proline; ROS—reactive oxygen species; TGZ—troglitazone.

The data suggest that TGZ-induced apoptosis in MDA-MB-231 cells cultured in medium without estradiol or deprived of ERβ is mediated by PRODH/POX and the process is facilitated by proline availability for PRODH/POX by TGZ-dependent inhibition of collagen biosynthesis (Scheme 2). It suggests that combined TGZ and anti-estrogen treatment could be considered in experimental therapy of ER negative breast cancers.

## Data Availability

The datasets used and/or analyzed during the current study are available from the corresponding author on reasonable request.

## Supplementary Materials

The following are available online at https://www.mdpi.com/article/10.3390/jcm10204641/s1, Figure S1: Representative blots of expression of PRODH/POX (POX) in MCF-7 and MDA-MB-231 cells; Figure S2: The PPAR expressions in MCF-7WT cells and MCF-7shPRODH/POX cells cultured in DMEM in the presence and absence of estradiol.; Figure S3. The PPAR expressions in MDA-MB-231WT cells and MDA-MB-231shPRODH/POX cells cultured in DMEM in the presence and absence of estradiol.; Figure S4: The AMPK expressions in MCF-7WT cells and MCF-7shPRODH/POX cells cultured in DMEM in the presence and absence of estradiol.; Figure S5: The AMPK expressions in MDA-MB-231WT cells and MDA-MB-231shPRODH/POX cells cultured in DMEM in the presence and absence of estradiol.; Figure S6: The PRODH/POX (POX) expressions in MCF-7WT cells and MCF-7shPRODH/POX cells cultured in DMEM in the presence and absence of estradiol.; Figure S7: The PRODH/POX (POX) expressions in MDA-MB-231WT cells and MDA-MB-231shPRODH/POX cells cultured in DMEM in the presence and absence of estradiol.; Figure S8: The non-cleaved-Caspase-3 expressions in MCF-7WT cells and MCF-7shPRODH/POX cells cultured in DMEM in the presence and absence of estradiol.; Figure S9: The cleaved-Caspase-3 expressions in MCF-7WT cells and MCF-7shPRODH/POX cells cultured in DMEM in the presence and absence of estradiol.; Figure S10: The PARP (upper bands) and cleaved-PARP (lower bands) expressions in MCF-7WT cells and MCF-7shPRODH/POX cells cultured in DMEM in the presence and absence of estradiol.; Figure S11: The non-cleaved-Caspase-9 expressions in MCF-7WT cells and MCF-7shPRODH/POX cells cultured in DMEM in the presence and absence of estradiol.; Figure S12: The cleaved-Caspase-9 expressions in MCF-7WT cells and MCF-7shPRODH/POX cells cultured in DMEM in the presence and absence of estradiol.; Figure S13: The p53 expressions in MCF-7WT cells and MCF-7shPRODH/POX cells cultured in DMEM in the presence and absence of estradiol.; Figure S14: The non-cleaved-Caspase-3 expressions in MDA-MB-231WT cells and MDA-MB-231shPRODH/POX cells cultured in DMEM in the presence and absence of estradiol.; Figure S15: The cleaved-Caspase-3 expressions in MDA-MB-231WT cells and MDA-MB-231shPRODH/POX cells cultured in DMEM in the presence and absence of estradiol.; Figure S16: The PARP and cleaved-PARP expressions in MDA-MB-231WT cells and MDA-MB-231shPRODH/POX cells cultured in DMEM in the presence and absence of estradiol.; Figure S17: The non-cleaved-Caspase-9 expressions in MDA-MB-231WT cells and MDA-MB-231shPRODH/POX cells cultured in DMEM in the presence and absence of estradiol.; Figure S18: The cleaved-Caspase-9 expressions in MDA-MB-231WT cells and MDA-MB-231shPRODH/POX cells cultured in DMEM in the presence and absence of estradiol.; Figure S19: The cleaved-Caspase-3 expressions in MDA-MB-231WT cells and MDA-MB-231shPRODH/POX cells cultured in DMEM in the presence and absence of estradiol.
