# Supplementary material for: Troglitazone-Induced PRODH/POX-Dependent Apoptosis Occurs in the Absence of Estradiol or ERβ in ER-Negative Breast Cancer Cells
Source: J Clin Med. 2021 Oct 10;10(20):4641. doi: 10.3390/jcm10204641 (PMC8538344; doi:10.3390/jcm10204641)
Supplement: Supplementary file 1 [file jcm-10-04641-s001.zip › jcm-1371195-supplementary.pdf]

Journal of
Clinical Medicine

Supplementary Material

Article

Troglitazone-Induced PRODH/POX-Dependent Apoptosis Occurs in the Absence of Estradiol or ERβ in ER-Negative Breast Cancer Cells

Sylwia Lewoniewska [1,†], Ilona Oscilowska [2,†], Thi Yen Ly Huynh [1], Izabela Prokop [1], Weronika Baszanowska [1], Katarzyna Bielawska [1] and Jerzy Palka [1,*]

[1] Department of Medicinal Chemistry, Medical University of Bialystok, Kilinskiego 1, 15-089 Bialystok, Poland; sylwialewoniewska123@wp.pl (S.L.); htyly79@gmail.com (T.Y.L.H.); izabela.prokop@umb.edu.pl (I.P.); w.baszanowska22@wp.pl (W.B.); katarzyna.bielawska@umb.edu.pl (K.B.)
[2] Department of Analysis and Bioanalysis of Medicines, Medical University of Bialystok, Kilinskiego 1, 15-089 Bialystok, Poland; ilona.zareba@gmail.com
* Correspondence: pal@umb.edu.pl; Tel.: +48-85-748-5706
† These authors contributed equally to this work.

1. Results

1.1. *Efficacy of shRNA-based PRODH/POX knock-down in MCF-7 and MDA-MB-231 cells*

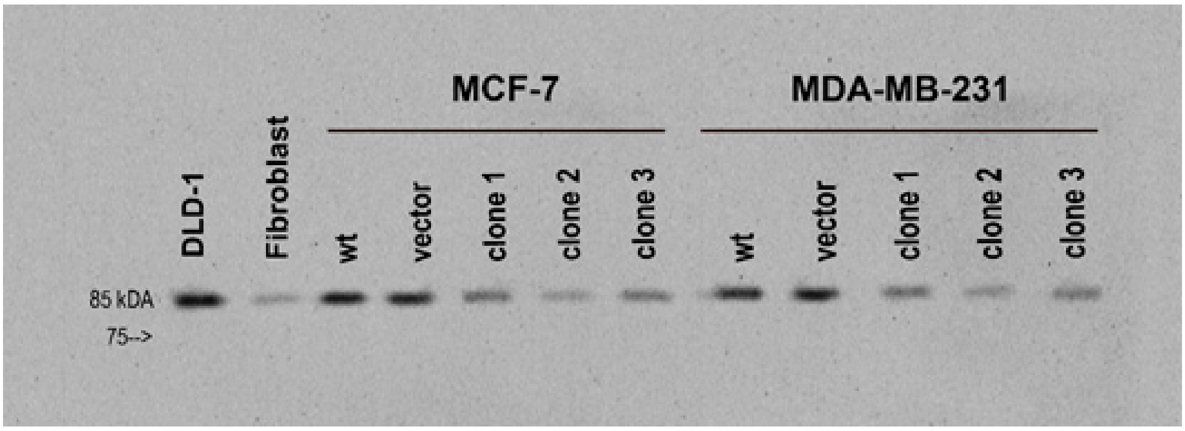

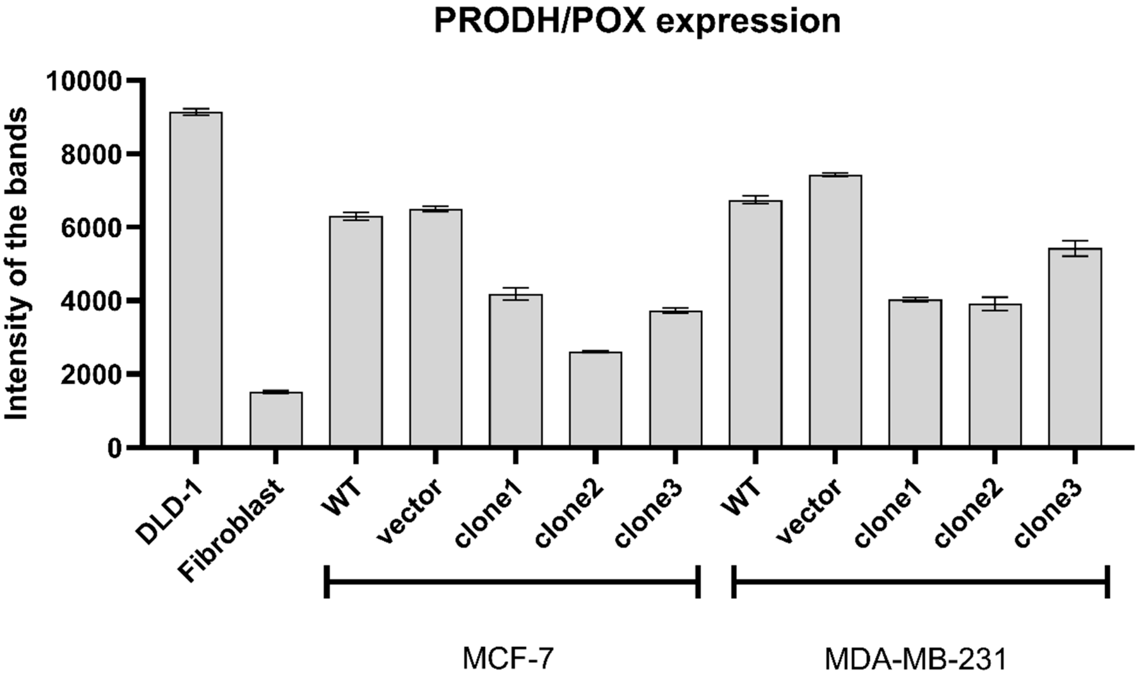

Figure S1. Representative blots of expression of PRODH/POX (POX) in MCF-7 and MDA-MB-231 cells. Transfection of the cells with different PRODH/POX shRNA constructs (clone1-3) were done. DLD-1 cells were used as a positive control and fibroblasts as a negative control for the expression of PRODH/POX. Representative gels of Western blotting and the intensity of POX bands was quantified by densitometry and normalized to GAPDH, values represent the mean (% of control) ± SD of three experiments, $*p < 0.001$.

1.2. *Western blots presented on Figure 2*

1.2.1. PPAR gamma

1.2.1.1. MCF-7 cell line

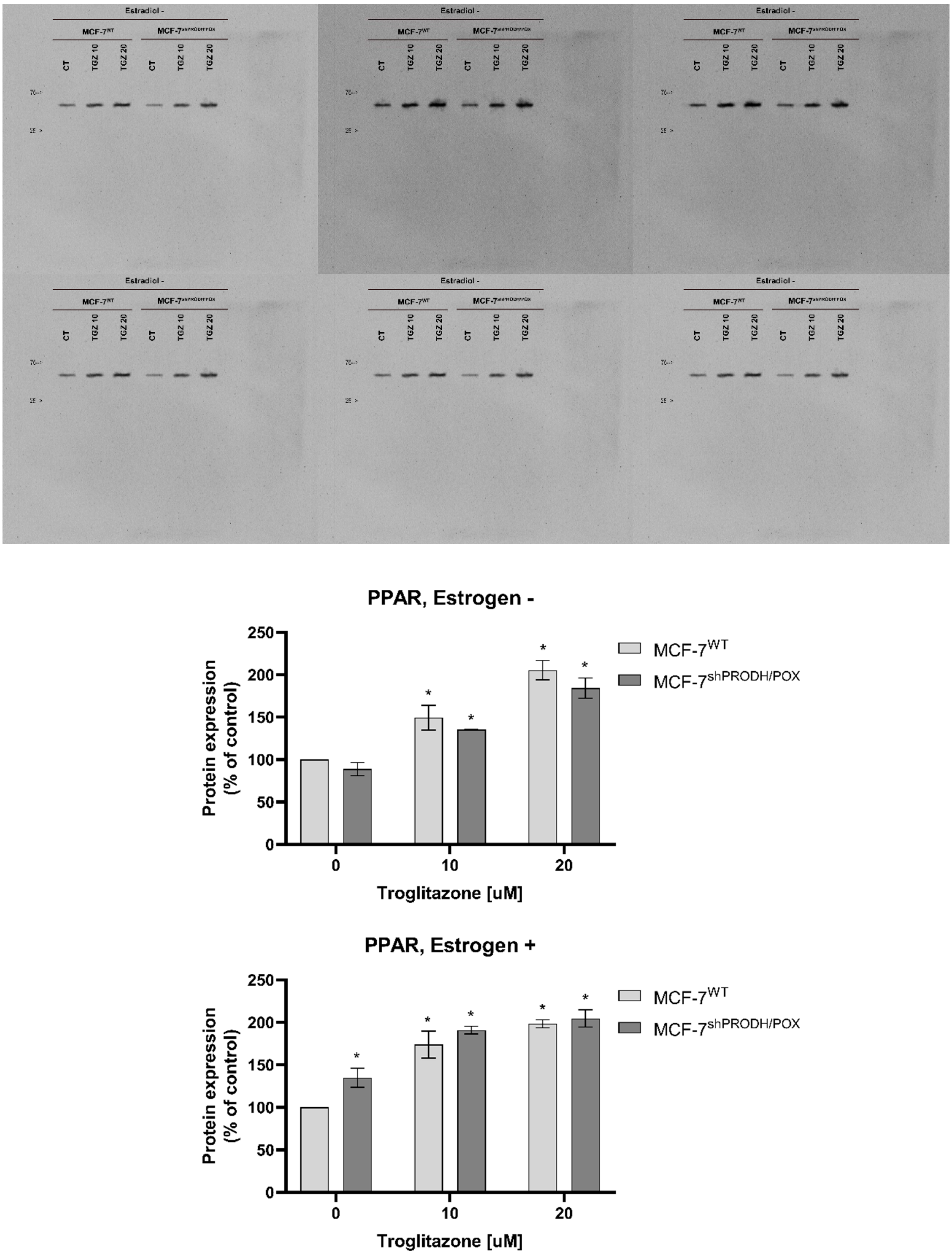

Figure S2. The PPAR expressions in MCF-7^{WT} cells and MCF-7$^{shPRODH/POX}$ cells cultured in DMEM in the presence and absence of estradiol. GAPDH expression was used as a loading control. The WB bands intensity of representative gels was quantified by densitometry and normalized to GAPDH. The densitometry values represent the mean (% of control) ± SD of three experiments, *$p < 0.001$.

1.2.1.2. MDA-MB-231 cell line

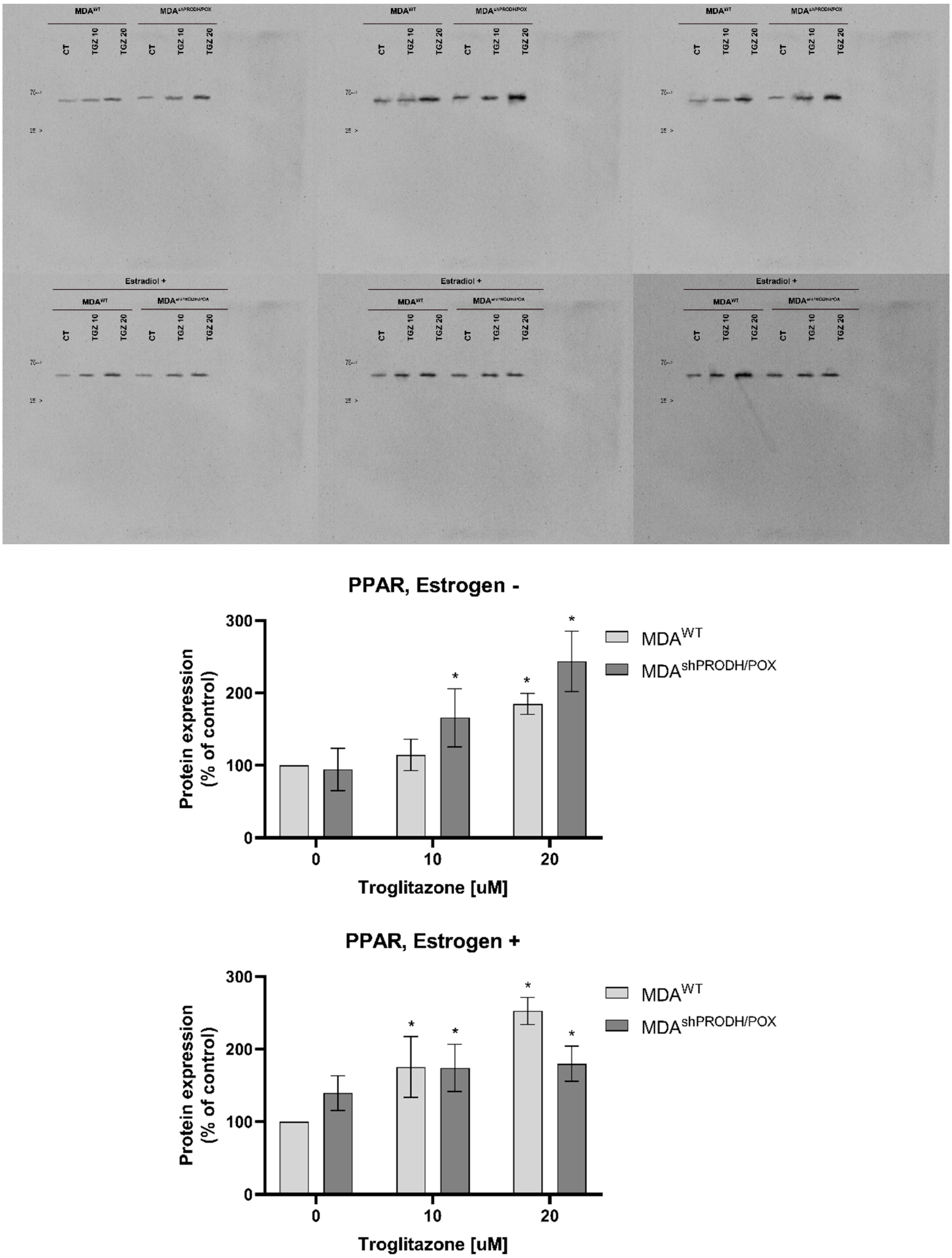

Figure S3. The PPAR expressions in MDA-MB-231^{WT} cells and MDA-MB-231$^{shPRODH/POX}$ cells cultured in DMEM in the presence and absence of estradiol. GAPDH expression was used as a loading control. The WB bands intensity of representative gels was quantified by densitometry and normalized to GAPDH. The densitometry values represent the mean (% of control) ± SD of three experiments, *$p < 0.001$.

1.2.2. AMPK alpha

1.2.2.1. MCF-7 cell line

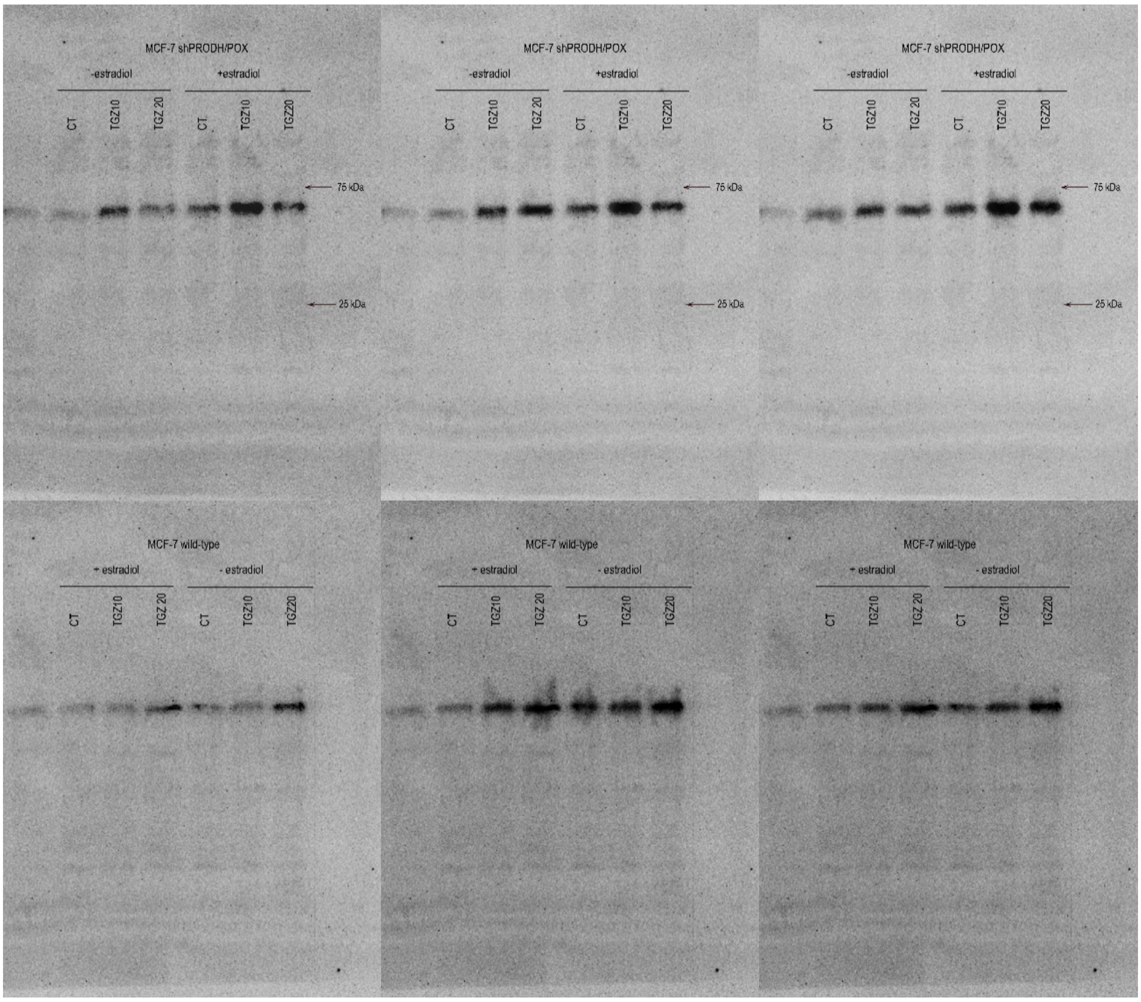

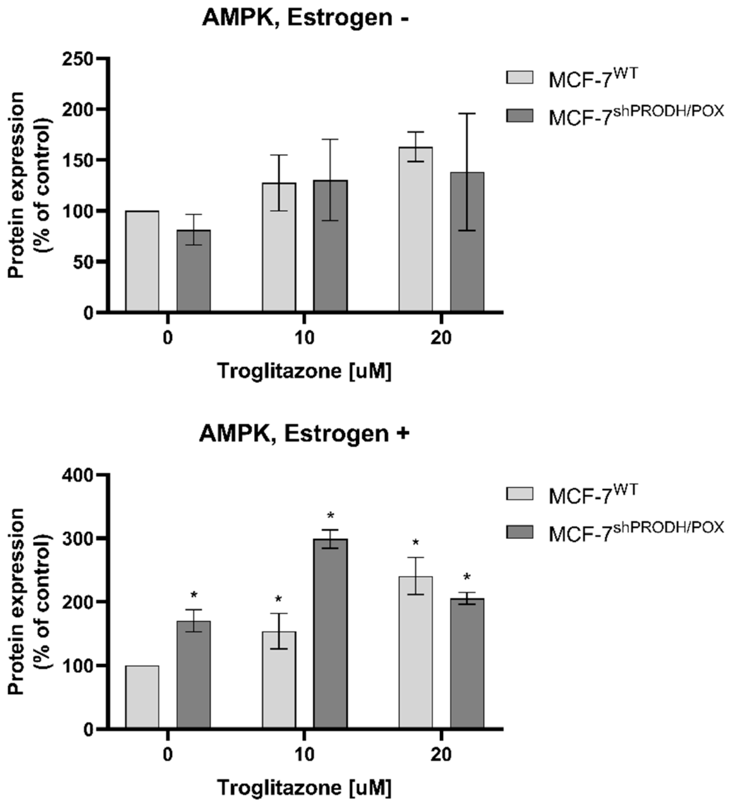

Figure S4. The AMPK expressions in MCF-7^{WT} cells and MCF-7$^{shPRODH/POX}$ cells cultured in DMEM in the presence and absence of estradiol. GAPDH expression was used as a loading control. The WB bands intensity of representative gels was quantified by densitometry and normalized to GAPDH. The densitometry values represent the mean (% of control) ± SD of three experiments, **p* < 0.001.

1.2.2.2. MDA-MB-231 cell line

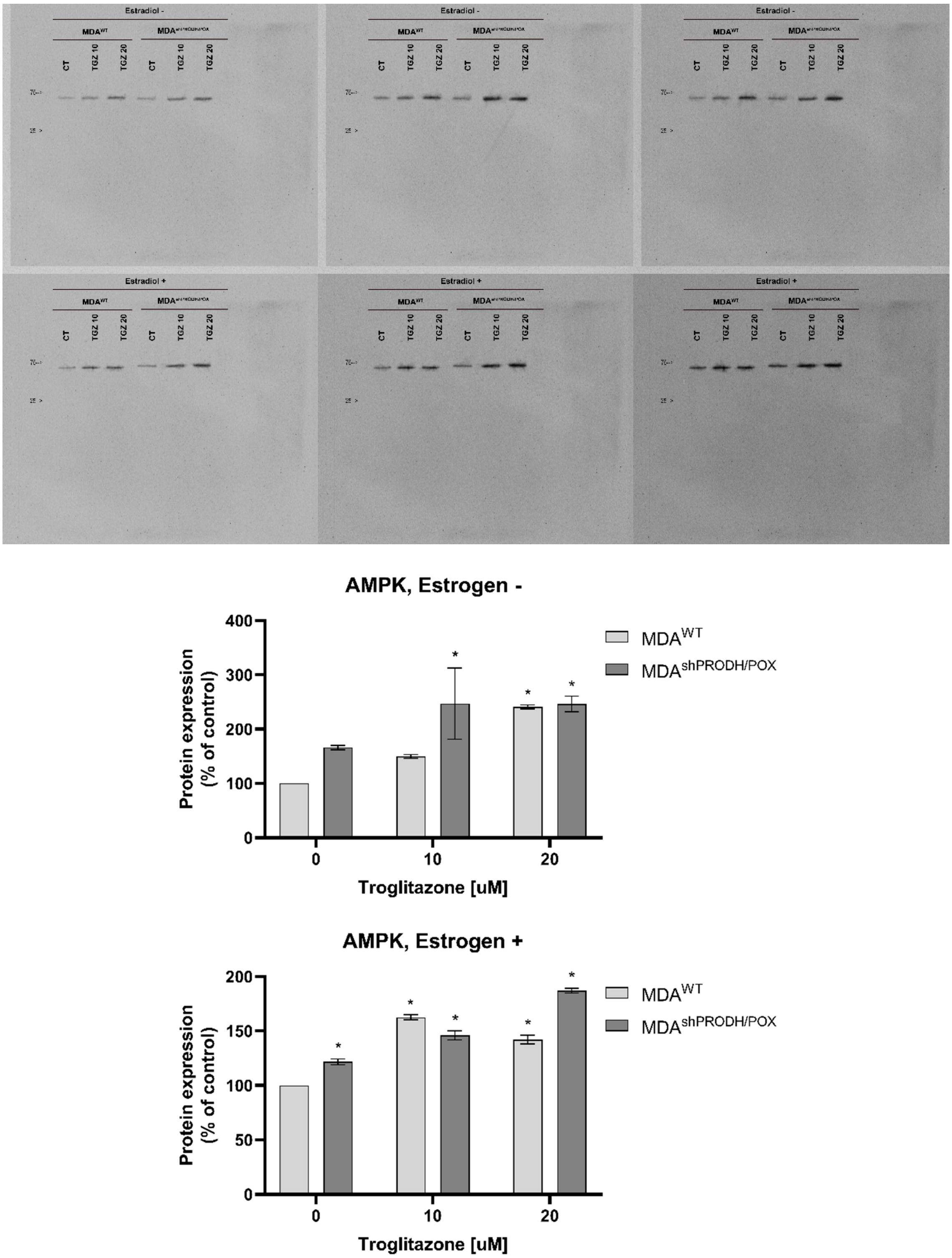

Figure S5. The AMPK expressions in MDA-MB-231^{WT} cells and MDA-MB-231$^{shPRODH/POX}$ cells cultured in DMEM in the presence and absence of estradiol. GAPDH expression was used as a loading control. The WB bands intensity of representative gels was quantified by densitometry and normalized to GAPDH. The densitometry values represent the mean (% of control) ± SD of three experiments, *$p < 0.001$.

1.2.3. PRODH/POX

1.2.3.1. MCF-7 cell line

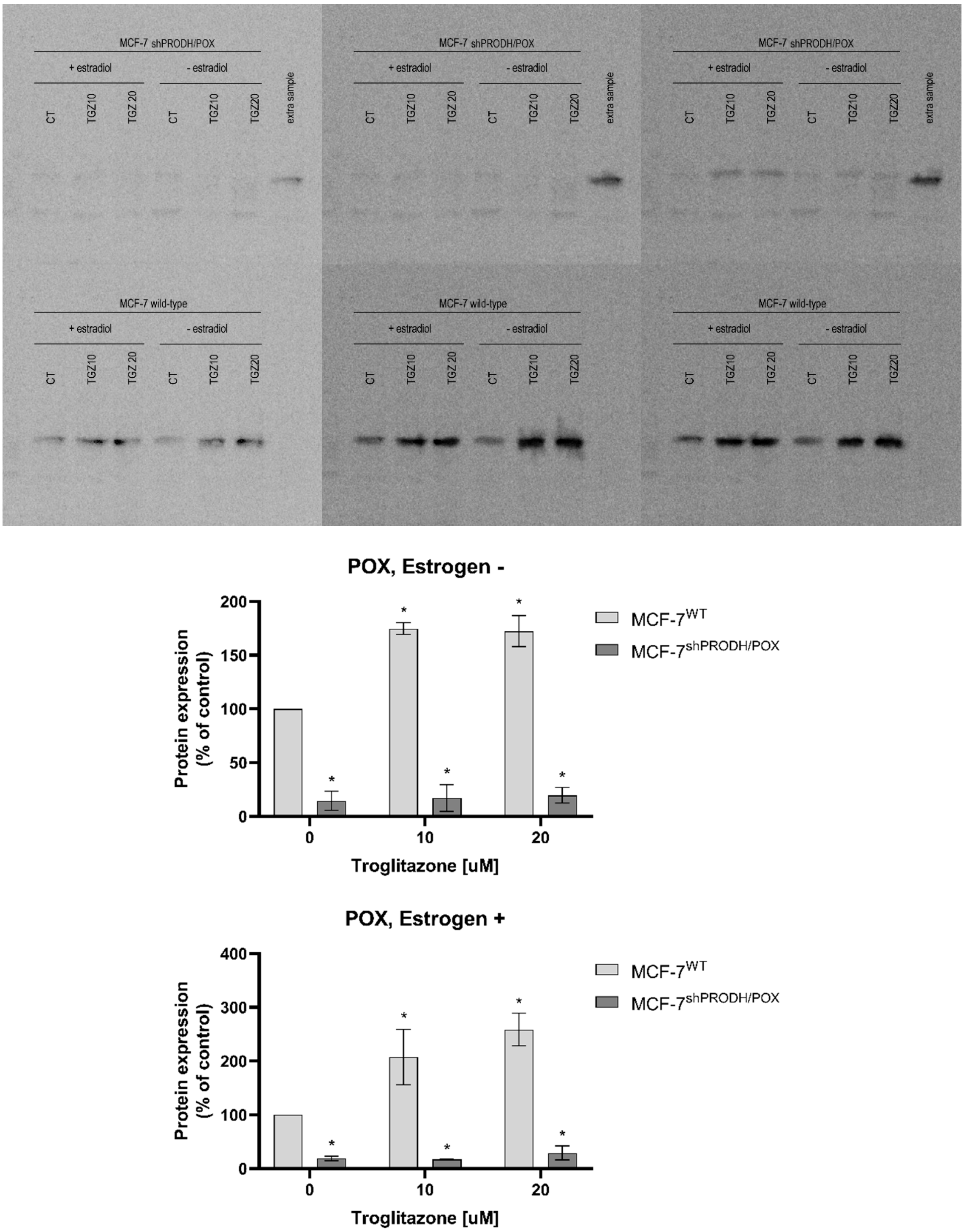

Figure S6. The PRODH/POX (POX) expressions in MCF-7^{WT} cells and MCF-7$^{shPRODH/POX}$ cells cultured in DMEM in the presence and absence of estradiol. GAPDH expression was used as a loading control. The WB bands intensity of representative gels was quantified by densitometry and normalized to GAPDH. The densitometry values represent the mean (% of control) ± SD of three experiments, **p* < 0.001.

1.2.3.2. MDA-MB-231 cell line

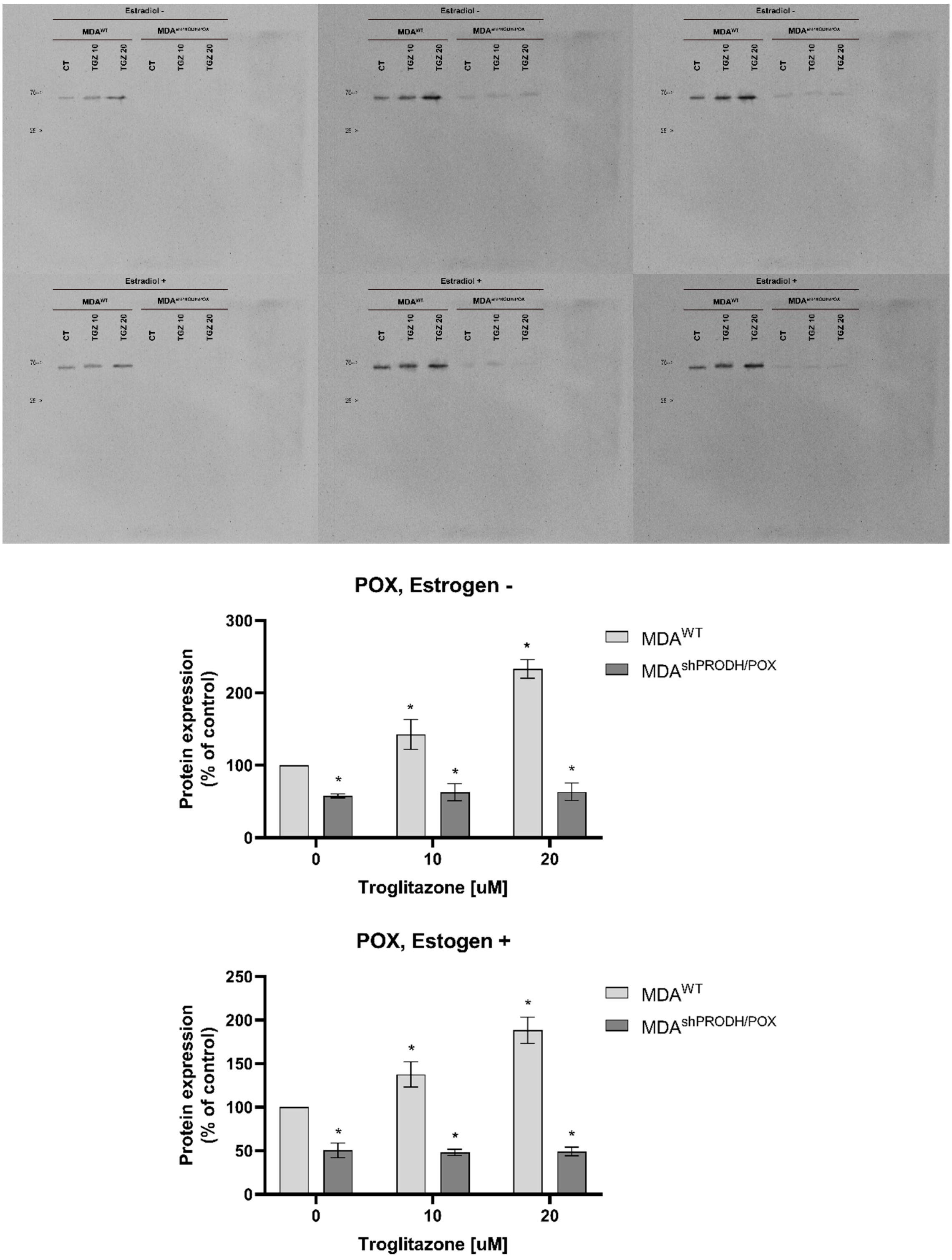

Figure S7. The PRODH/POX (POX) expressions in MDA-MB-231^{WT} cells and MDA-MB-231$^{shPRODH/POX}$ cells cultured in DMEM in the presence and absence of estradiol. GAPDH expression was used as a loading control. The WB bands intensity of representative gels was quantified by densitometry and normalized to GAPDH. The densitometry values represent the mean (% of control) ± SD of three experiments, *$p < 0.001$.

1.3. *Western blots presented on Figure 3*

1.3.1. *Caspase-3*

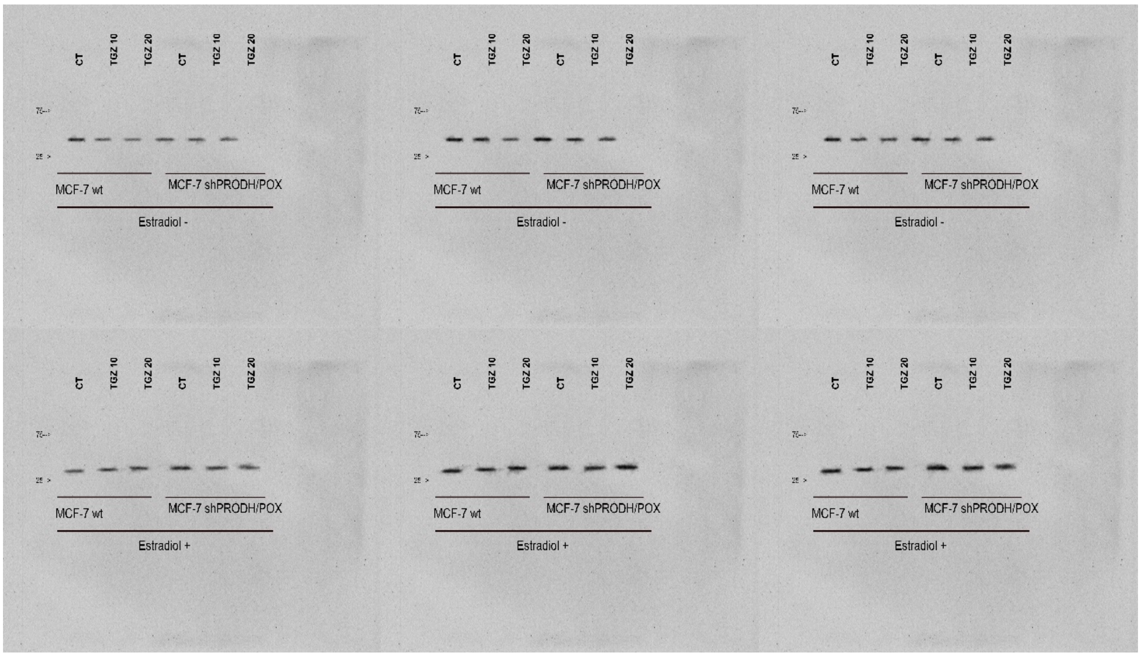

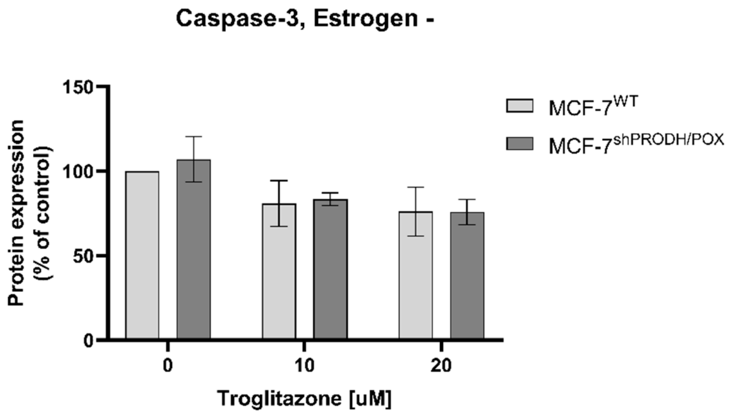

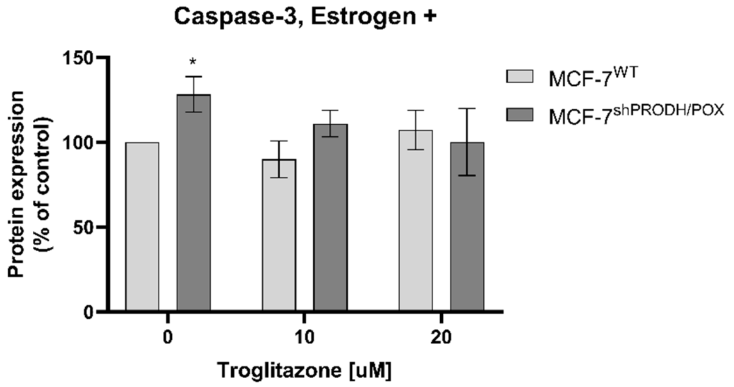

Figure S8. The non-cleaved-Caspase-3 expressions in MCF-7^{WT} cells and MCF-7$^{shPRODH/POX}$ cells cultured in DMEM in the presence and absence of estradiol. GAPDH expression was used as a loading control. The WB bands intensity of representative gels was quantified by densitometry and normalized to GAPDH. The densitometry values represent the mean (% of control) ± SD of three experiments, **p* < 0.001.

1.3.2. *Cleaved-Caspase-3*

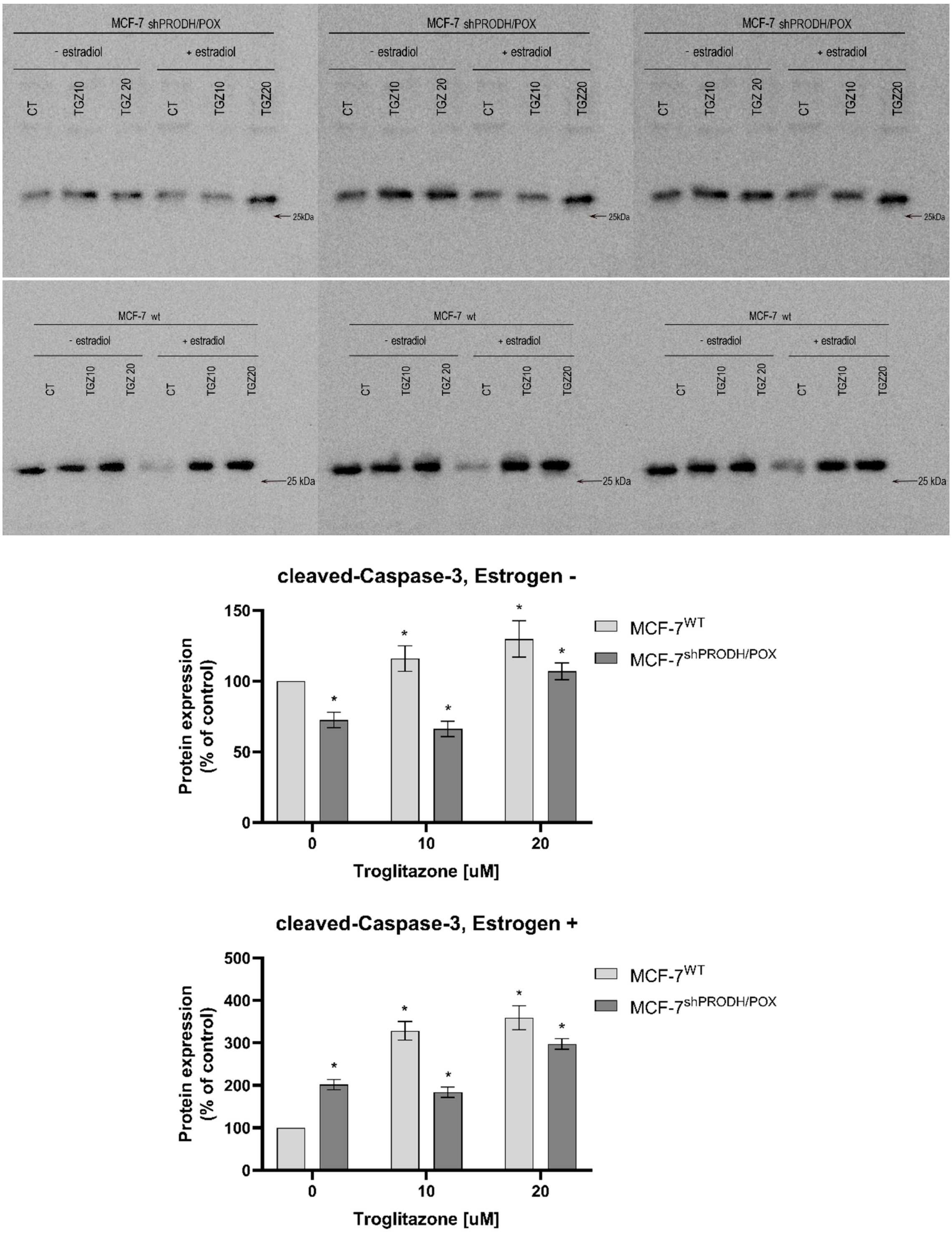

Figure S9. The cleaved-Caspase-3 expressions in MCF-7^{WT} cells and MCF-7$^{shPRODH/POX}$ cells cultured in DMEM in the presence and absence of estradiol. GAPDH expression was used as a loading control. The WB bands intensity of representative gels was quantified by densitometry and normalized to GAPDH. The densitometry values represent the mean (% of control) ± SD of three experiments, *$p < 0.001$.

1.3.3. PARP and cleaved-PARP

PARP (upper bamds) and cleaved-PARP (lower bands)

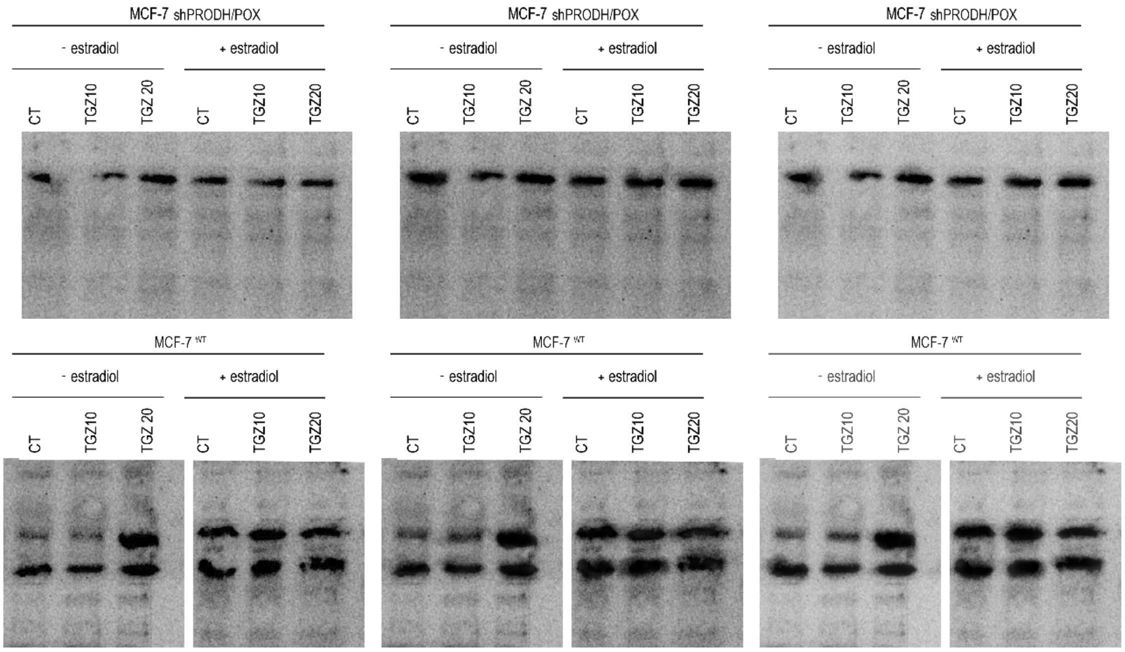

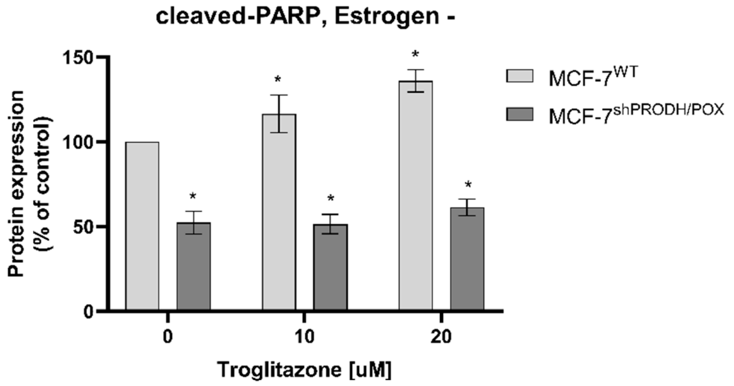

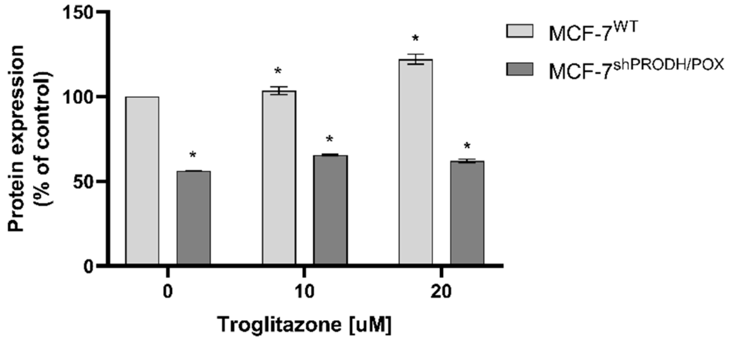

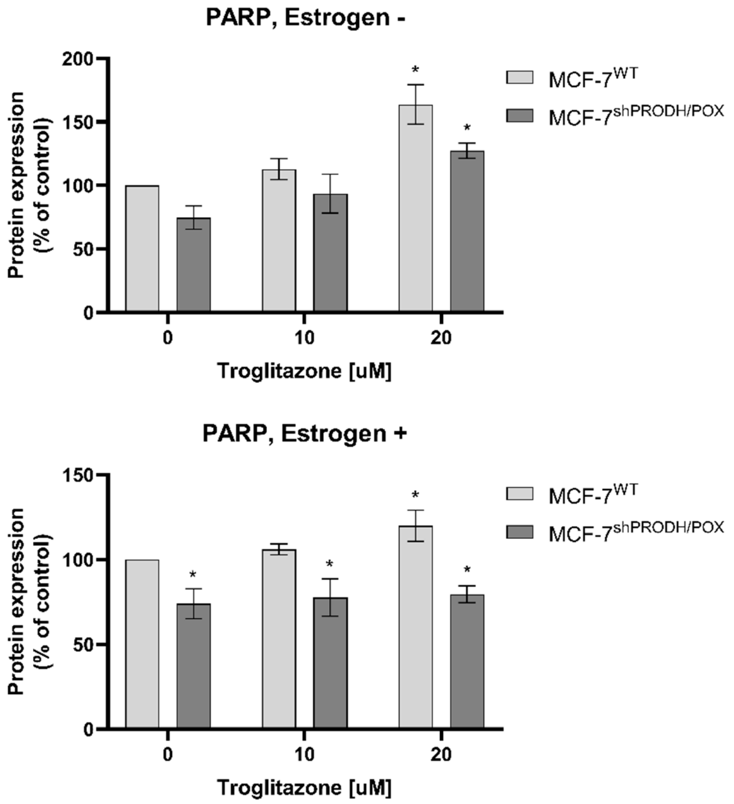

Figure S10. The PARP (upper bands) and cleaved-PARP (lower bands) expressions in MCF-7^{WT} cells and MCF-7$^{shPRODH/POX}$ cells cultured in DMEM in the presence and absence of estradiol. GAPDH expression was used as a loading control. The WB bands intensity of representative gels was quantified by densitometry and normalized to GAPDH. The densitometry values represent the mean (% of control) ± SD of three experiments, $*p < 0.001$.

1.3.4. *Caspase-9*

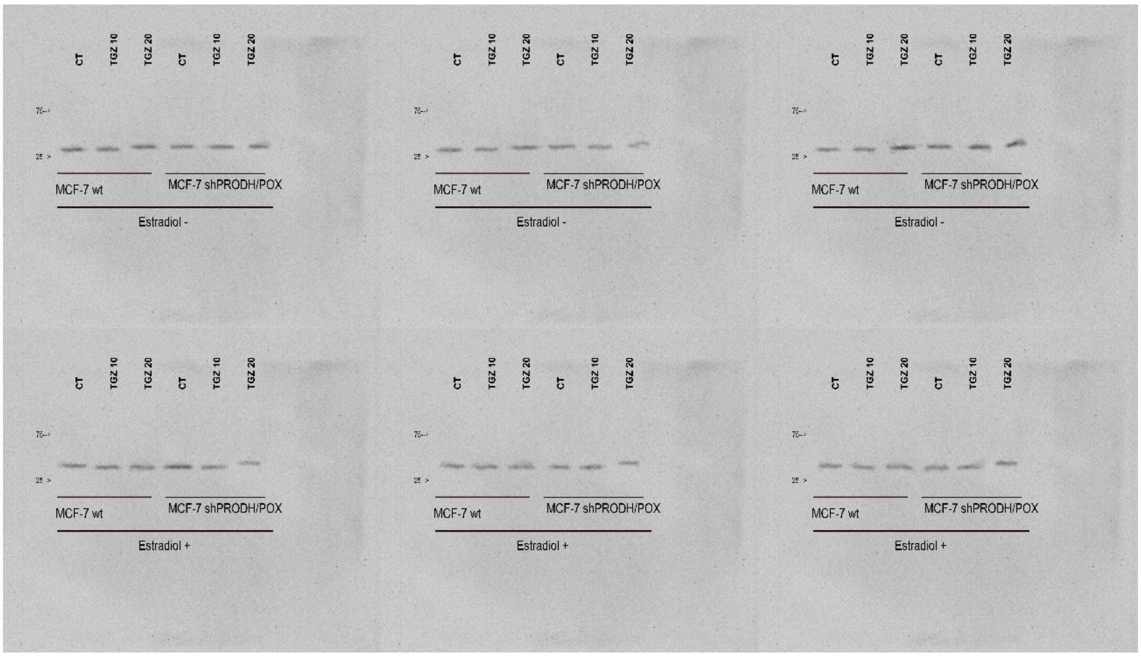

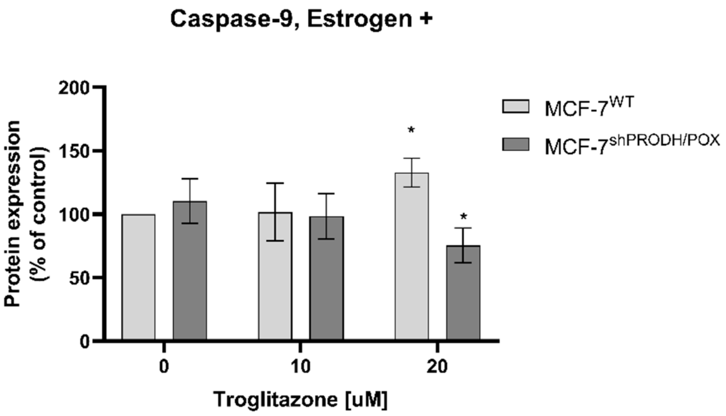

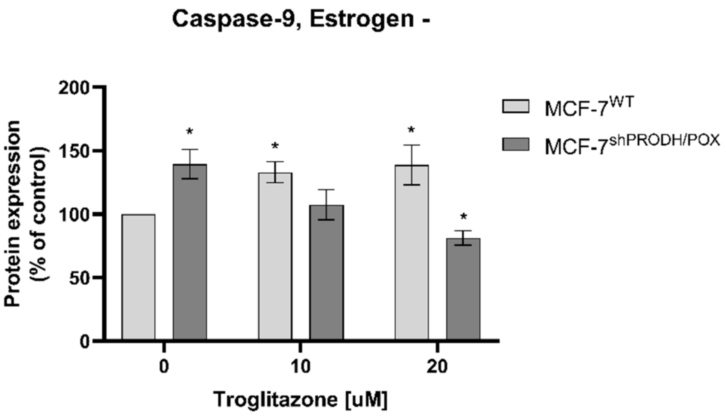

Figure S11. The non-cleaved-Caspase-9 expressions in MCF-7^{WT} cells and MCF-7$^{shPRODH/POX}$ cells cultured in DMEM in the presence and absence of estradiol. GAPDH expression was used as a loading control. The WB bands intensity of representative gels was quantified by densitometry and normalized to GAPDH. The densitometry values represent the mean (% of control) ± SD of three experiments, *p < 0.001.

1.3.5. *Cleaved-Caspase-9*

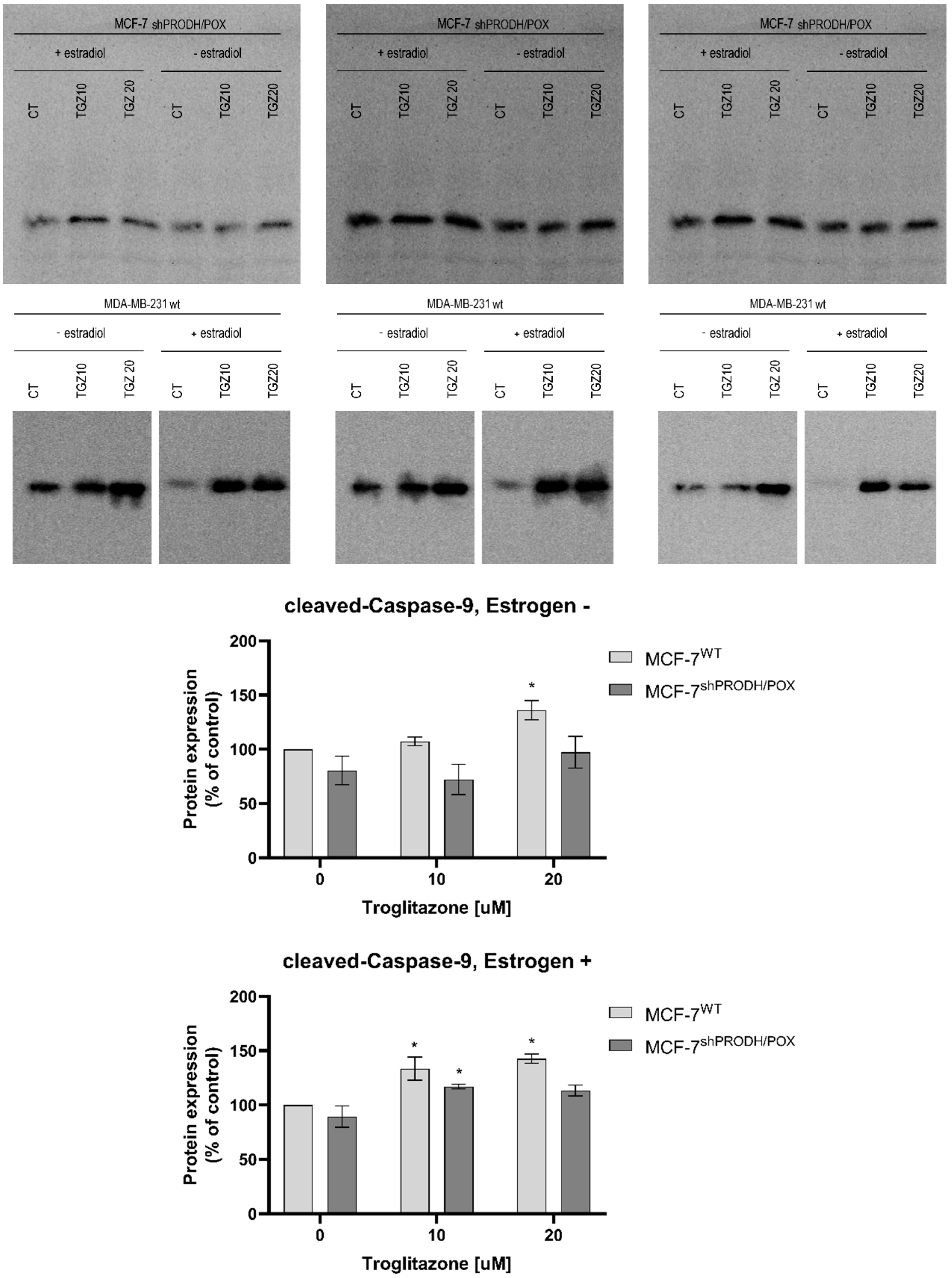

Figure S12. The cleaved-Caspase-9 expressions in MCF-7^{WT} cells and MCF-7$^{shPRODH/POX}$ cells cultured in DMEM in the presence and absence of estradiol. GAPDH expression was used as a loading control. The WB bands intensity of representative gels was quantified by densitometry and normalized to GAPDH. The densitometry values represent the mean (% of control) ± SD of three experiments, *p < 0.001.

1.3.6. *p53*

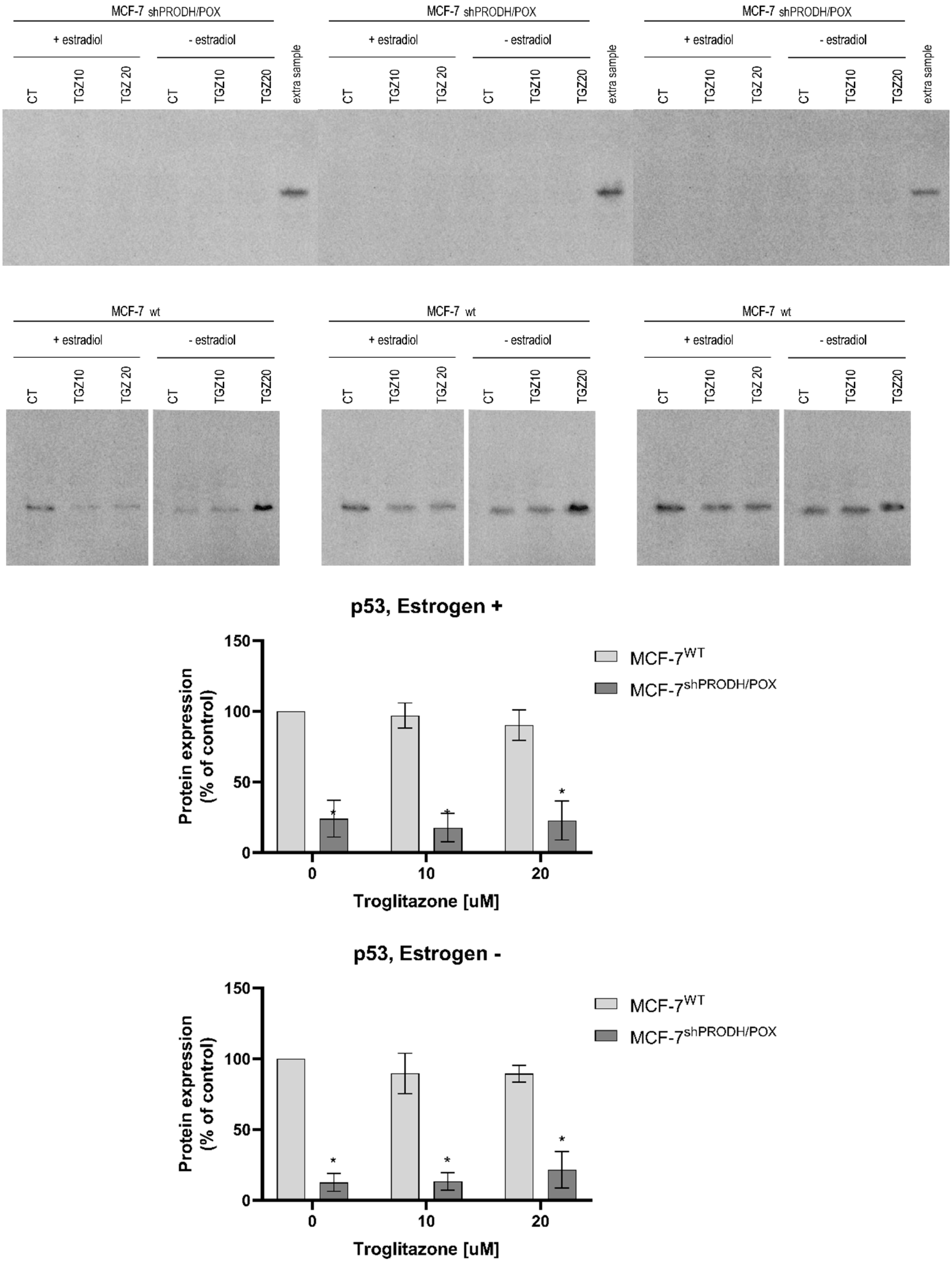

Figure S13. The p53 expressions in MCF-7^{WT} cells and MCF-7$^{shPRODH/POX}$ cells cultured in DMEM in the presence and absence of estradiol. GAPDH expression was used as a loading control. The WB bands intensity of representative gels was quantified by densitometry and normalized to GAPDH. The densitometry values represent the mean (% of control) ± SD of three experiments, *$p < 0.001$.

1.4. *Western blots presented on Figure 5*

1.4.1. *Caspase-3*

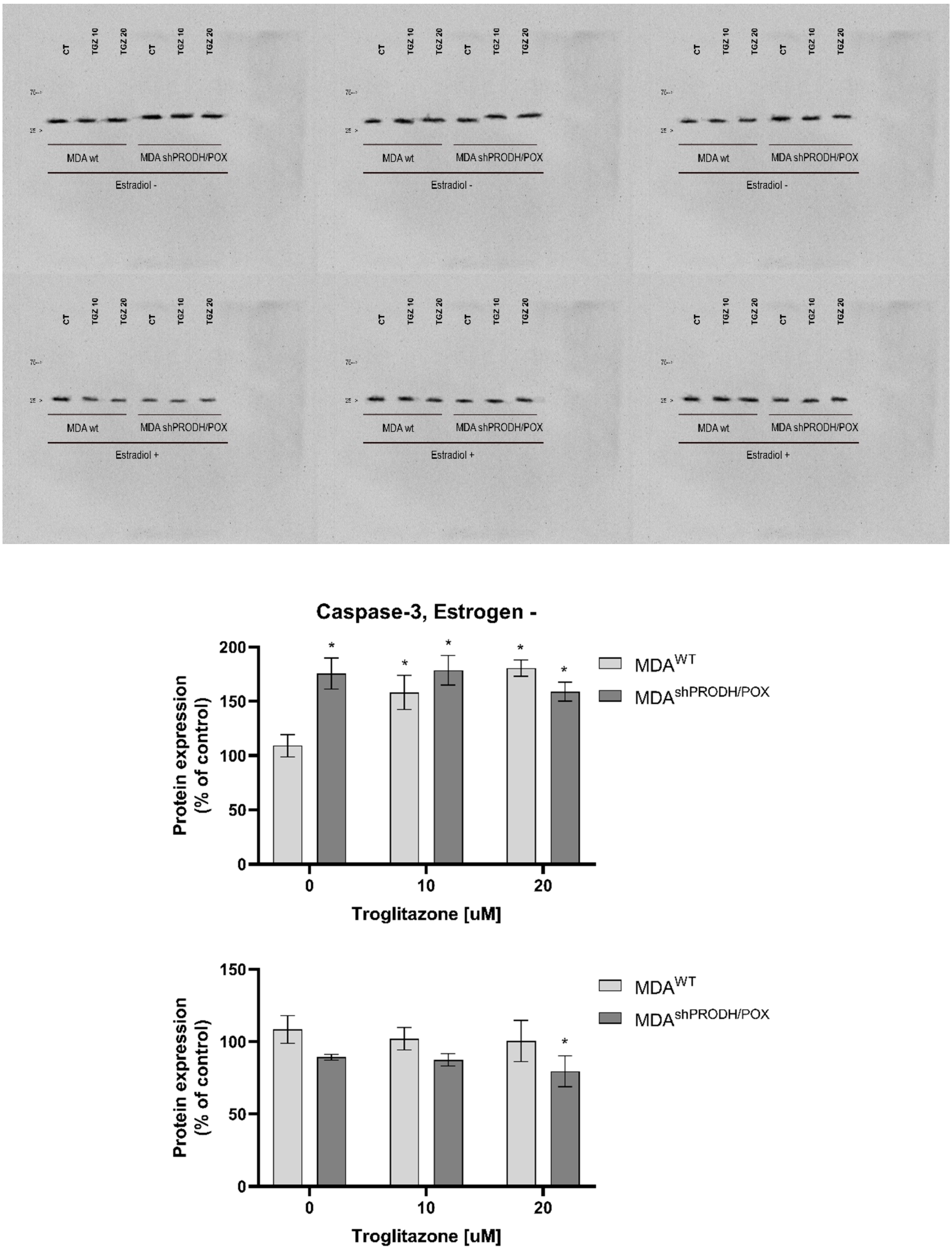

Figure S14. The non-cleaved-Caspase-3 expressions in MDA-MB-231^{WT} cells and MDA-MB-231$^{shPRODH/POX}$ cells cultured in DMEM in the presence and absence of estradiol. GAPDH expression was used as a loading control. The WB bands intensity of representative gels was quantified by densitometry and normalized to GAPDH. The densitometry values represent the mean (% of control) ± SD of three experiments, *$p < 0.001$.

1.4.2. *Cleaved-Caspase-3*

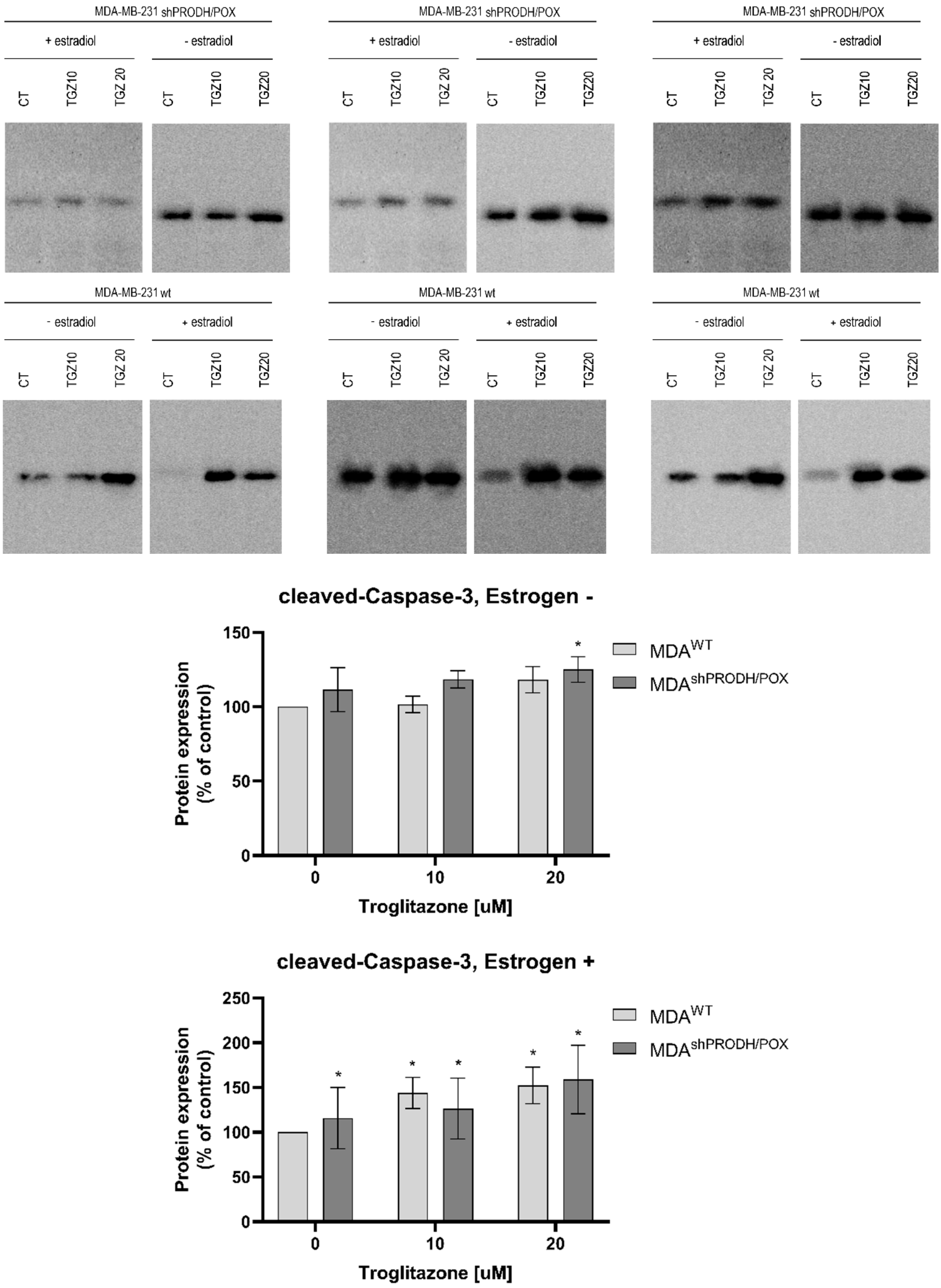

Figure S15. The cleaved-Caspase-3 expressions in MDA-MB-231^{WT} cells and MDA-MB-231$^{shPRODH/POX}$ cells cultured in DMEM in the presence and absence of estradiol. GAPDH expression was used as a loading control. The WB bands intensity of representative gels was quantified by densitometry and normalized to GAPDH. The densitometry values represent the mean (% of control) ± SD of three experiments, *p < 0.001.

1.4.3. *PARP and cleaved-PARP*

PARP (upper bamds) and cleaved-PARP (lower bands)

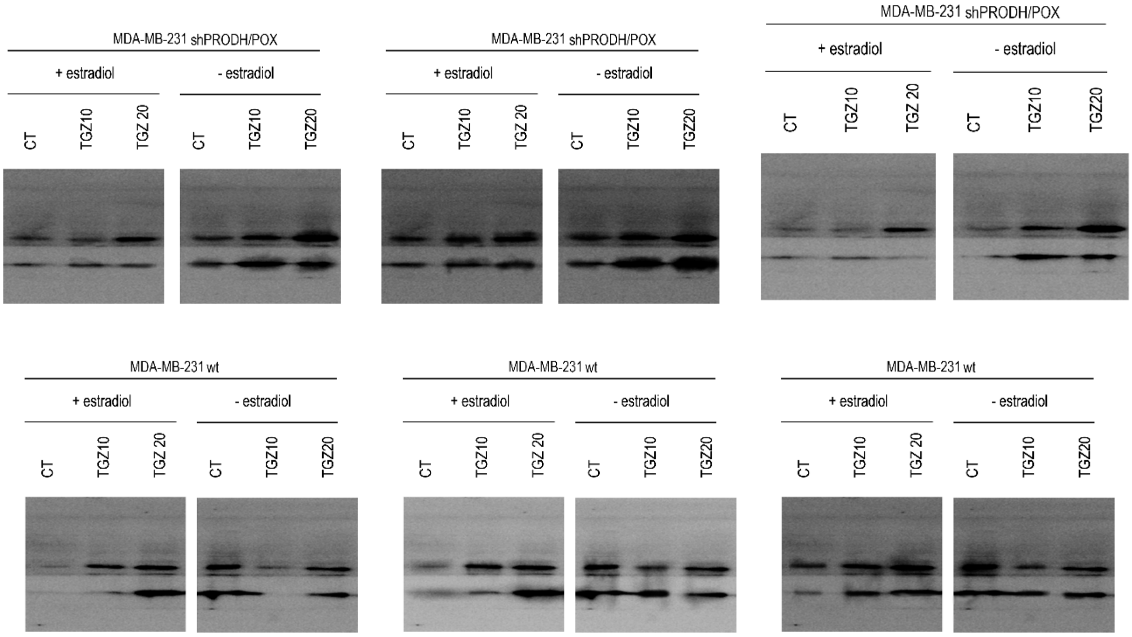

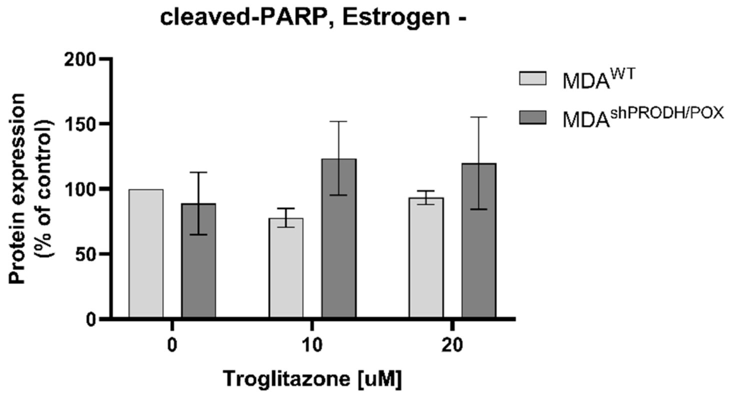

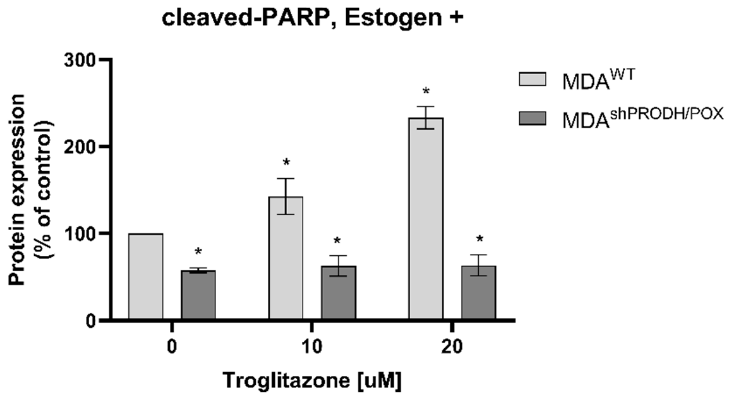

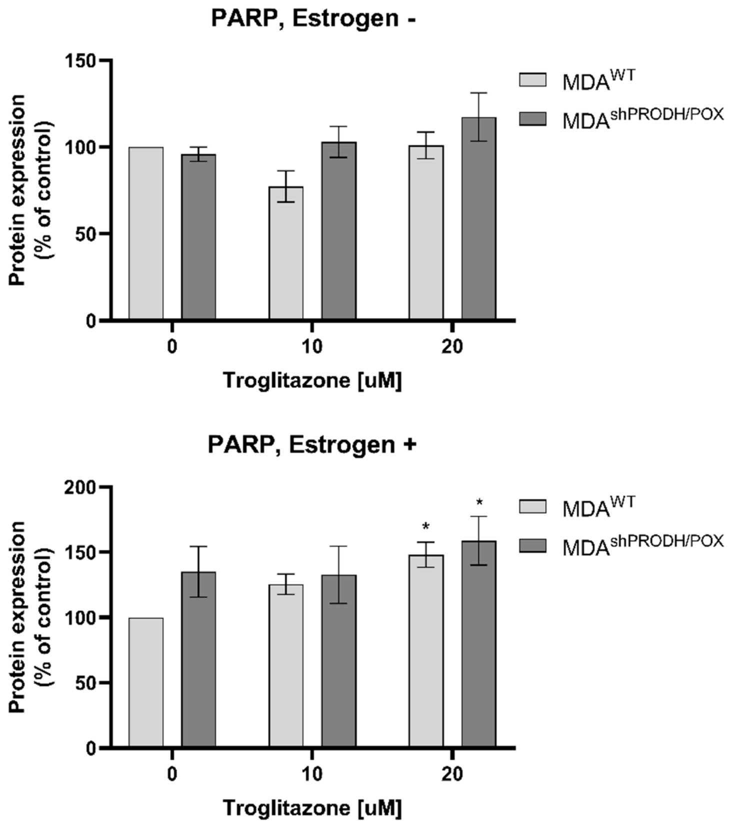

Figure S16. The PARP and cleaved-PARP expressions in MDA-MB-231^{WT} cells and MDA-MB-231$^{shPRODH/POX}$ cells cultured in DMEM in the presence and absence of estradiol. GAPDH expression was used as a loading control. The WB bands intensity of representative gels was quantified by densitometry and normalized to GAPDH. The densitometry values represent the mean (% of control) ± SD of three experiments, $^{*}p < 0.001$.

1.4.4. *Caspase-9*

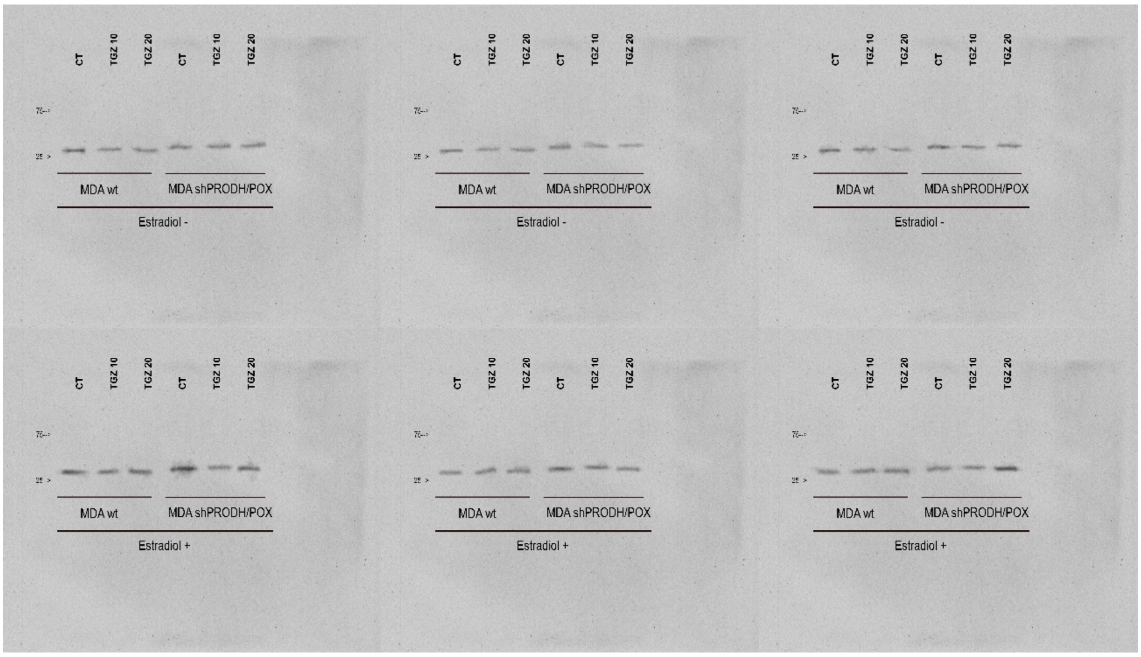

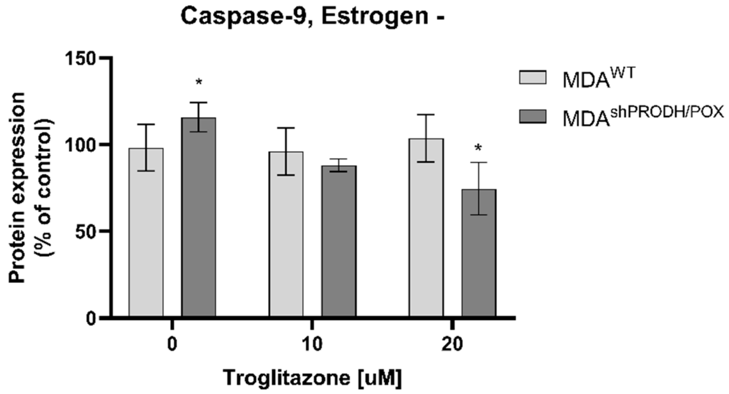

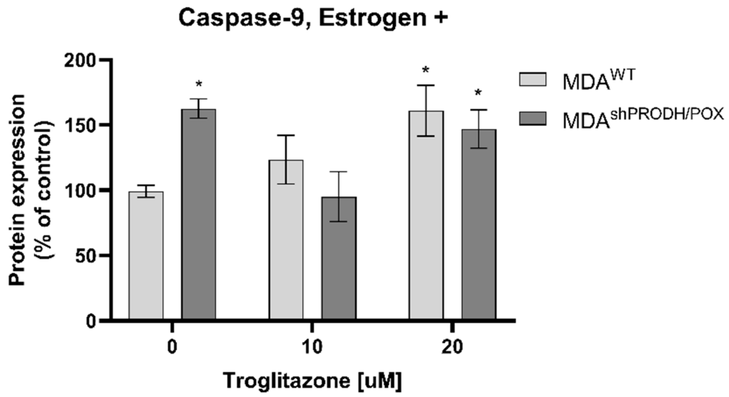

Figure S17. The non-cleaved-Caspase-9 expressions in MDA-MB-231^{WT} cells and MDA-MB-231$^{shPRODH/POX}$ cells cultured in DMEM in the presence and absence of estradiol. GAPDH expression was used as a loading control. The WB bands intensity of representative gels was quantified by densitometry and normalized to GAPDH. The densitometry values represent the mean (% of control) ± SD of three experiments, *$p < 0.001$.

1.4.5. *Cleaved-Caspase-9*

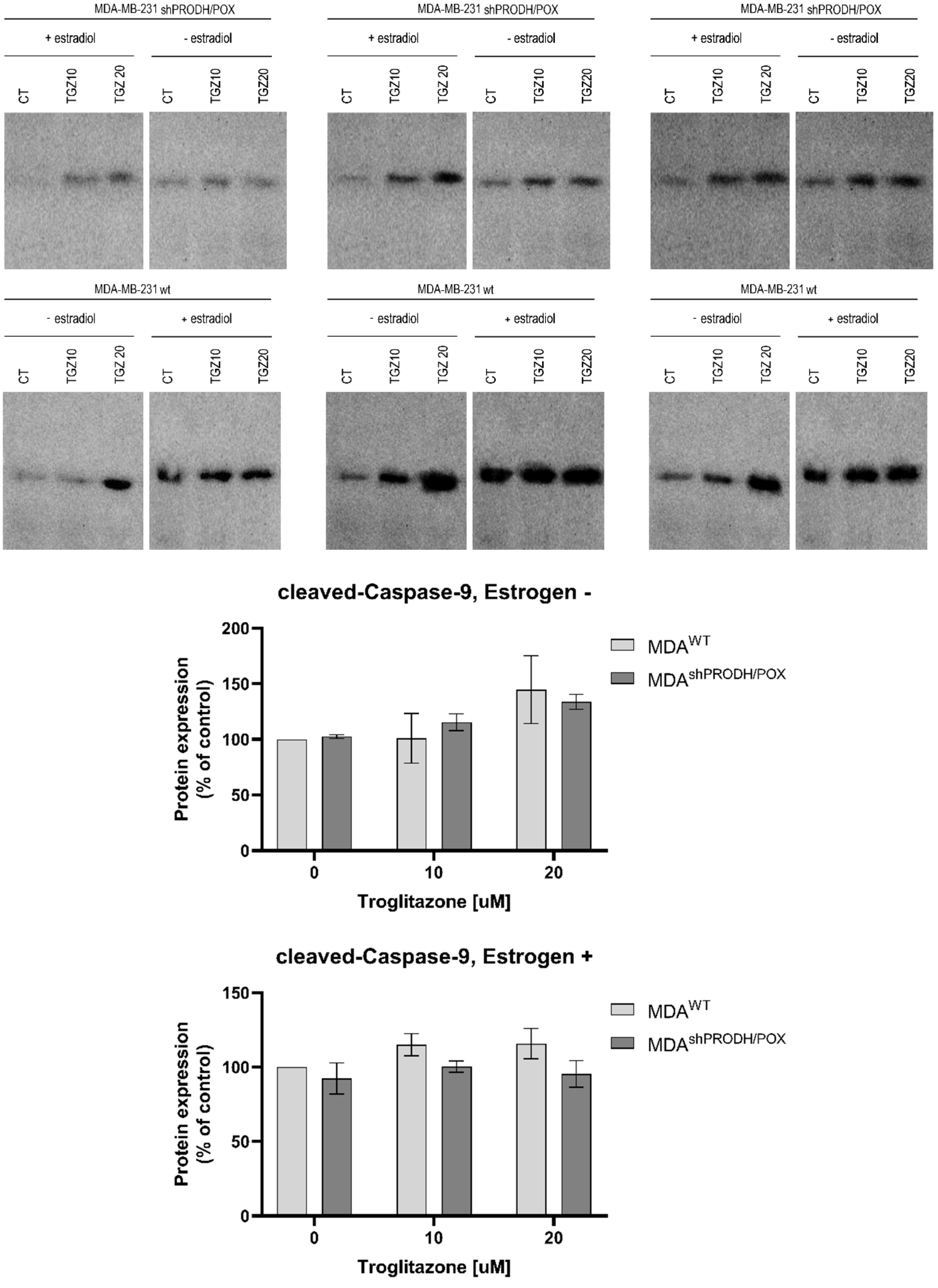

Figure S18. The cleaved-Caspase-9 expressions in MDA-MB-231^{WT} cells and MDA-MB-231$^{shPRODH/POX}$ cells cultured in DMEM in the presence and absence of estradiol. GAPDH expression was used as a loading control. The WB bands intensity of representative gels was quantified by densitometry and normalized to GAPDH. The densitometry values represent the mean (% of control) ± SD of three experiments, **p* < 0.001.

1.4.6. *p53*

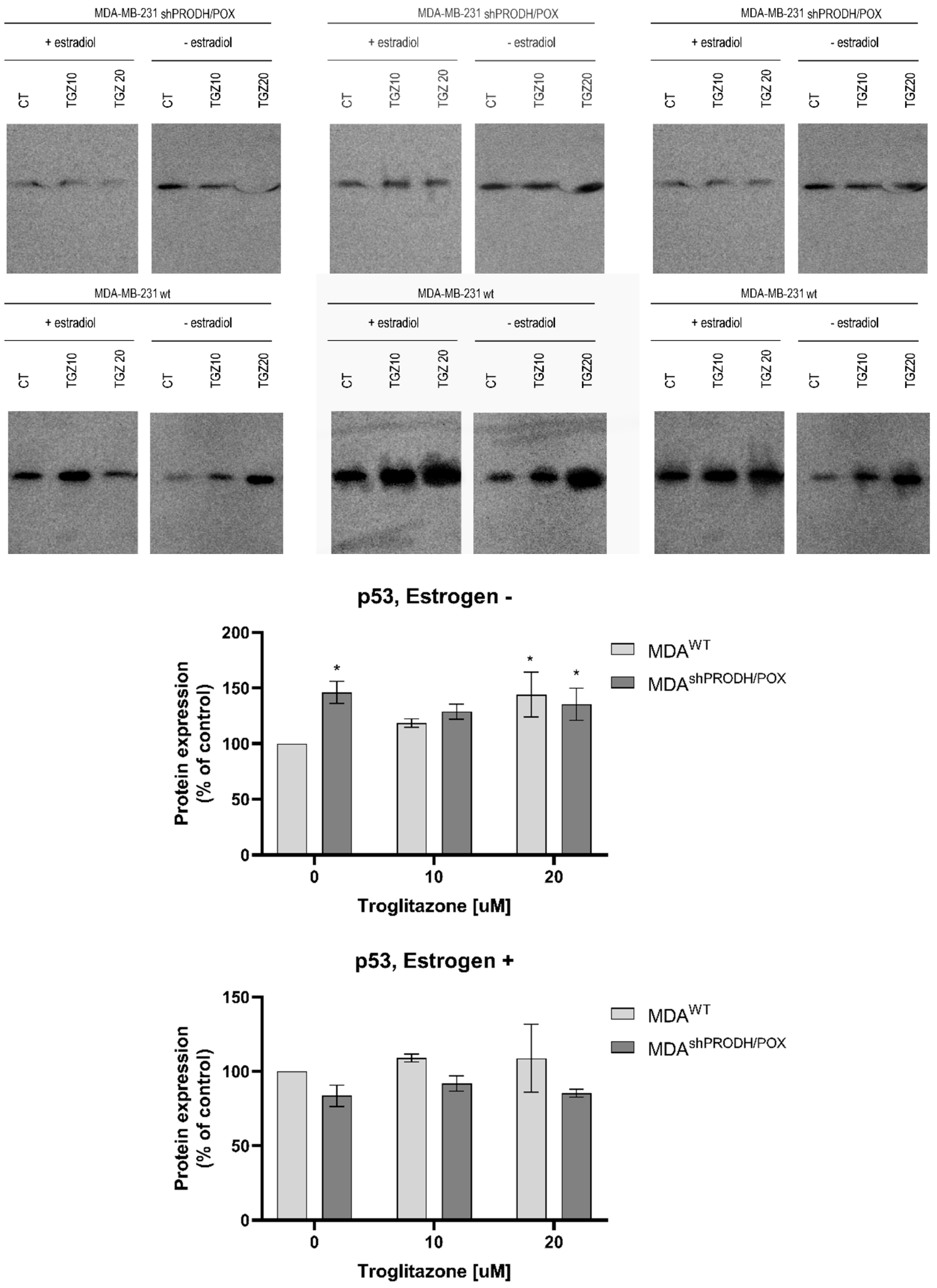

Figure S19. The cleaved-Caspase-3 expressions in MDA-MB-231^{WT} cells and MDA-MB-231$^{shPRODH/POX}$ cells cultured in DMEM in the presence and absence of estradiol. GAPDH expression was used as a loading control. The WB bands intensity of representative gels was quantified by densitometry and normalized to GAPDH. The densitometry values represent the mean (% of control) ± SD of three experiments, *p < 0.001.
